# Metabolic licensing at the dendritic cell–NK cells immune synapse in viral asthma exacerbations

**DOI:** 10.3389/fimmu.2026.1798209

**Published:** 2026-04-02

**Authors:** Yan-Jiao Chen, Gabriel Shimizu Bassi, Yong-Qing Yang

**Affiliations:** 1 Research Institute of Acupuncture and Meridian, Yueyang Hospital of Integrated Traditional Chinese and Western Medicine, Shanghai University of Traditional Chinese Medicine, Shanghai, China; 2 School of Rehabilitation Science, Shanghai University of Traditional Chinese Medicine, Shanghai, China

**Keywords:** asthma, autophagy, dendritic cells, immunometabolism, natural killer cells, viral infection

## Abstract

Asthma exacerbations are predominantly triggered by respiratory viral infections, yet current therapies largely fail to restore effective antiviral immunity. Emerging data indicate that this failure is tightly coupled to dysregulated immunometabolism within the asthmatic lung. This review advances the concept of a dendritic cell–natural killer (DC–NK) metabolic checkpoint, whereby the metabolic state of DCs, regulated by autophagy and AMPK/mTOR signaling, licenses NK cells for antiviral effector function. In type 2-high, type 2−low, and obesity-related asthma endotypes, chronic hypoxia, HIF−1α stabilization, ORMDL3–ceramide signaling, and systemic metabolic stress converge to induce highly glycolytic, Th2/Th17−polarizing DCs in a lactate-rich, acidic microenvironment. We propose that these DCs modulate NK cell metabolism through three interlinked axes: (i) cytokine-mediated metabolic licensing (IL−12, IL−15, IL−18), (ii) exosome-mediated delivery of activating versus metabolically suppressive cargo, and (iii) intense perisynaptic nutrient competition that depletes local glucose while lactate accumulation and acidosis further inhibit NK cell function. The result is a “double metabolic hit” that renders lung-resident NK cells metabolically exhausted, IFN−γ−deficient, and unable to clear virally infected targets despite preserved cytotoxic machinery. Although many mechanistic insights derive from murine and *in vitro* models, converging human metabolomic, genetic, and functional data support this framework and define clear research gaps. If validated in human studies, targeting the DC-NK cell metabolic checkpoint with agents that restore autophagic plasticity, rebalance AMPK/mTOR signaling, or normalize airway nutrient and pH landscapes may represent a promising strategy to prevent viral-triggered asthma exacerbations.

## 1 Introduction

Asthma affects over 300 million individuals worldwide and is defined by chronic airway inflammation, reversible airflow obstruction, and bronchial hyperresponsiveness (1). Current treatments manage daily symptoms, but asthmatic exacerbations accounts for most related illness, death, and healthcare expenses. In the United States alone, asthma-related costs exceed $80 billion annually, with viral-induced hospitalizations driving the majority of this burden (1). Up to 85% of exacerbations are triggered by respiratory viral infections, predominantly human rhinovirus (HRV), influenza, and respiratory syncytial virus (RSV) (2, 3). Standard treatments like inhaled corticosteroids and bronchodilators reduce inflammation but compromise antiviral defences, leaving patients susceptible to repeated viral-induced episodes (4–8). Despite extensive research into the clinical consequences of viral-induced exacerbations, the immunopathological mechanisms underlying failed antiviral responses in asthma remain incompletely understood.

Early hypotheses implicated intrinsic deficiencies in interferon (IFN) production by the airway epithelium (4), but newer evidence indicates a dysfunction in innate immunity, specifically involving dendritic cells (DCs) and natural killer (NK) cells responses (9). NK cells isolated from asthmatic patients exhibit reduced cytotoxicity, low IFN-γ production, and metabolic exhaustion (10–12), while airway DCs display maladaptive activation states, promoting Th2/Th17 polarization rather than antiviral responses (13–16). These observations suggest a critical dysfunction within the DC–NK cell axis. However, to our knowledge, no prior review has systematically examined DC-NK cell metabolic “checkpoint” controlling asthma exacerbation. Here, we propose a metabolic, DC-centric checkpoint model licensing NK cells for antiviral competence in asthma. Although our framework is applicable to all respiratory RNA viruses (including HRV, RSV, and influenza), the strongest support comes from HRV and RSV models, and extending this to severe acute respiratory syndrome coronavirus 2 or viral-bacterial coinfections remains testable. Additionally, human evidence supporting NK cell metabolic dysfunction in severe asthma is robust, but direct quantification of DCs autophagy flux and cytokine production in human asthmatic airways during viral infection has not been performed (10–12).

On this basis, we formulate three testable hypotheses supported by murine models, *in vitro* data, and limited human observational studies. First, asthmatic DCs exhibit compromised autophagy during viral infections [distinct from allergen-induced basal autophagy (17)], suppressing IL-12/IL-15/IL-18 production and NK cell metabolic licensing. Second, high-glycolytic immune cells create a glucose-deficient, lactate-rich airway microenvironment, starving lung-resident NK cells, particularly at the DC-NK cell immune-synapse. Third, therapeutic restoration of DCs autophagy can normalize NK cell metabolic competence and reduce viral exacerbations (9, 18–21). Furthermore, this review integrates three levels of evidence: (1) Established facts from primary human studies [e.g., NK cell functional deficits in asthma (10–12), elevated airway lactate (22, 23)]; (2) Mechanistic principles from primary experimental studies (e.g., glycolysis requirement for NK cell cytotoxicity (20, 21), autophagy-dependent cytokine secretion in DCs (24); and (3) Hypothetical integration proposing that these mechanisms converge into a DC-NK cell metabolic checkpoint. We also explicitly indicate when extrapolating from non-asthma contexts or proposing untested connections.

## 2 The metabolic landscape of the asthmatic lung

The asthmatic airways are characterized by chronic inflammation, structural remodelling, and persistent exposure to pathogens, promoting severe metabolic disturbances, such as hypoxia, acidosis, nutrient depletion, and waste accumulation (23, 25, 26), and creating a highly competitive environment for vital resources like glucose, cytokines, and oxygen (25, 27, 28). Persistent tissue hypoxia stabilizes the hypoxia−inducible factor−1α (HIF−1α) in bronchial epithelial cells, airway smooth muscle, and infiltrating immune cells, inducing a Warburg−like metabolic shift that emphasizes cell glycolysis over mitochondrial oxidative phosphorylation (OXPHOS) (25, 28). This shift reduces pyruvate flux into the mitochondria and upregulates glucose transporter type 1 (GLUT1) and multiple glycolytic enzymes, elevating glucose uptake, generating lactate as byproduct, and acidifying the airways (22, 25, 28). This creates a hostile environment for immune surveillance and inflammation control.

Clinical findings demonstrate reduced pH in exhaled breath condensates from asthmatic patients, especially during acute exacerbations (29–31), indicating airway acidification and lactate accumulation. Mechanistically, continuous ATP breakdown for glycolysis promotes adenosine buildup in the airways, stimulating further bronchoconstriction and Th2 cytokines production (32). Additionally, persistent allergen exposure generates uric acid, triggering Th2 cell polarization and inflammation via Syk and PI3K−δ signalling (33). These local metabolic disturbances are also linked to systemic acid–base abnormalities. For instance, respiratory acidosis caused by CO_2_ retention often correlates with metabolic and lactic acidosis from tissue hypoxia and muscle fatigue, especially during severe exacerbations (34). The resultant systemic hypoxia and acidosis reinforce HIF−1α signaling and glycolytic shift in lung−resident DCs and NK cells (34–36), establishing a continuous cycle of Th2 inflammation (25, 28) and antiviral dysfunction (10) that aggravate airway obstruction. Beyond metabolic reprogramming, hypoxia promotes complex effects on immune cell trafficking. For instance, hypoxia promotes endothelial ICAM-1 expression (37) while compromising NK cells migration through altered chemokine receptor signaling and reduced responsiveness to inflammatory gradients (35). Furthermore, hypoxia upregulates CXCR4 expression on CD56^bright^ NK cells (38), but the overall impact on NK cell recruitment to inflamed airways during viral infections remains context-dependent and incompletely understood (see NK Cells and Glycolysis Dependence topic). This acidosis is observed even in stable asthma, with patients exhibiting higher serum and sputum lactate levels than healthy controls (22, 23, 39), indicating metabolic dysregulation and permanent “nutrient pressure” at baseline. Here, highly glycolytic DCs intensifies nutrient sequestration, starving NK cells (13–15) and promoting a hostile acidic environment at the NK-DC synapse that further suppress IFN−γ production and antiviral immunity (40, 41). However, lactate accumulation likely reflects multiple sources, including β−agonists (type B lactic acidosis), excessive respiratory muscle work and fatigue (34), and bulk glycolysis from infiltrating immune cells (22). The relative contribution of DCs glycolysis to total airway lactate remains unquantified, representing a key knowledge gap for therapeutic targeting (see Critical Research Gaps and Future Directions Topic).

Metabolomic analyses of sputum and bronchoalveolar lavage fluid (BALF) from asthmatic patients demonstrate lactate accumulation (22, 23), consistent with glycolytic signatures correlated with inflammation severity in preclinical models, yet direct causal relationships in humans remain to be established. Additionally, human data also indicate systemic mechanistic target of rapamycin (mTOR) activation in acute exacerbations, confirming preclinical evidence suggesting metabolic constraints and limited immune responses (26), although direct causal links in humans are not yet fully established. These findings are consistent with a proposed “metabolic checkpoint” model for effective antiviral response in airways NK cells, potentially regulated by DCs metabolism via autophagy and the AMP−activated protein kinase (AMPK)/mTOR pathways, though direct demonstration in human asthma is still required. Targeting these specific mechanistic nodes could rescue antiviral competence and prevent asthma exacerbations. Within this metabolically constrained environment, DC autophagy emerges as a potential regulator of antiviral competence, though direct human validation remains limited.

## 3 Dendritic cells autophagic machinery and metabolic support

Asthmatic DCs undergo a metabolic shift from OXPHOS and fatty acid oxidation to aerobic glycolysis, accompanied by increased glutamine catabolism, enhanced lipid synthesis, and elevated mitochondrial itaconate production. This metabolic reprogramming is tightly linked to autophagy, a required process for cellular survival during metabolic shifts (42–44). The general requirement for autophagy in DC maturation is well-established via ATG5-deletion studies (43, 44), and the switch from OXPHOS to glycolysis in activated DCs is confirmed *ex vivo (*45). However, a critical gap remains: whether asthmatic DCs exhibit deficient viral-induced secretory autophagy (distinct from basal, allergen-regulated autophagy) is currently only supported by murine models of house dust mite (HDM)/RSV (17, 24) and has not been proven using isolated human airway DCs during exacerbations. Upstream signals, including allergen exposure (HDM), activation of pattern recognition receptors (Toll-like receptors (TLR)s, Dectin-1), thymic stromal lymphopoietin (TSLP) and airway cytokines, such as including granulocyte-macrophage colony-stimulating factor (GM-CSF) produced by structural, immune, and epithelial cells, coordinate this integrated metabolic and autophagic responses (17, 39, 46). Here, HIF-1α stabilization and mTOR complex 1 (mTORC1) signaling operate as key factors for supporting glycolysis and suppressing autophagy in DCs (26, 28, 47, 48). mTORC1 suppresses autophagy, stimulating protein synthesis (26, 48), while HIF-1α and itaconate induce mitophagy, clearing faulty mitochondria, and attenuating ROS production (49, 50). These metabolic adaptations promote antigen presentation, co-stimulatory molecules, and cytokine secretion, stimulating Th2/Th17 polarization and suppressing regulatory T-cell (Treg) phenotypes (17, 45). However, recent evidence demonstrates that allergen (HDM) exposure upregulates autophagy in DCs, promoting DC maturation, and Th2/Th17 polarization (17), challenging our model. Resolving this apparent contradiction requires comparative scRNAseq analysis of airway DCs during allergen exposure vs. active viral infection in the same asthmatic patients, examining stimulus-specific autophagy pathway activation (canonical vs. non-canonical ATG machinery) and downstream functional outputs. The apparent contradiction may originate from different mechanisms. Primary explanations include the divergent pathways between allergen-induced (potentially degradative) vs. viral-induced (non-degradative) autophagy (24). Furthermore, we must consider the temporal and cellular context: the kinetic difference of allergen-responsive CD11b^+^ DCs contrasting virus-stimulated cDC1 (51, 52). Finally, the outcome itself differs, with Th2 polarization controlled by allergens and NK cell licensing regulated by viral stimuli. Importantly, asthma exacerbations involve both chronic allergen and acute viral inflammation, and whether asthmatic DCs exhibit excessive basal autophagy (allergen-induced, pro-inflammatory) alongside deficient secretory autophagy (viral-responsive, antiviral) remains untested in humans. This dual dysregulation could explain persistent inflammation with failed viral clearance. Downstream of DCs dysfunction, NK cells, as the primary cytotoxic effectors against viral infection, exhibit metabolic vulnerabilities that may explain checkpoint failure.

This review focuses on conventional DC (cDC) subsets as the primary mediators of the proposed checkpoint because cDC1 and cDC2 form direct immune synapses with NK cells, exhibit well-characterized metabolic profiles during allergen and viral responses, and constitute the structural substrate for the direct cellular axes of IL-15 trans-presentation, exosome-mediated cargo transfer, and perisynaptic nutrient competition described herein (18, 51–53). However, pDCs are important upstream modulators of this axis and deserve special consideration. pDC-derived type I IFNs serve a dual function: it directly operates on NK cells via IFNAR to stimulate cytotoxic function, while indirectly conditioning cDCs for IL-15 trans-presentation (53–57). In asthma, this pDC contribution is compromised at multiple levels: pDC numbers are reduced in asthmatic airways (58), their IFN-α output is suppressed by FcϵRI/IgE cross-linking (55), and the lactate-rich asthmatic microenvironment may further inhibit pDC IFN-I production through direct suppression via lactate-sensing GPR81 signaling and post-activation LDHB downregulation (59). These mechanisms converge to suppress upstream type I IFN signal upon which optimal cDC-mediated NK cell licensing depends on. An “IFN-low” pDC phenotype has been independently associated with childhood asthma (60), supporting the clinical relevance of this deficit. While pDC dysfunction exacerbates checkpoint failure by attenuating upstream licensing signals, the checkpoint mechanism itself is defined by the direct perisynaptic metabolic events between cDC1/cDC2 and NK cells (e.g., glucose competition, cytokine trans-presentation, and exosome transfer). Currently, an equivalent metabolic characterization of pDC within human asthmatic airways is unavailable.

## 4 NK cells and glycolysis dependence

The asthmatic lung microenvironment, characterized by glucose depletion, hypoxia, and acidosis, alongside intense nutrient competition from Th2 and DCs, promote severe metabolic strain in NK cells, compromising their function. This section explores how the asthmatic lung imposes a “starved” metabolic state on NK cells and its subsequent impact on disease progression.

Upon activation, resting NK cells transition from exclusively OXPHOS to increasing both glycolysis and OXPHOS through mTORC1 HIF-1α signalling (26, 61). Glycolysis provides rapid ATP generation and generates key intermediates for *de novo* synthesis of effector molecules, like granzyme B, perforin, and IFN-γ (21), while increased OXPHOS ensures the long-term, high-volume energy supply required for effector functions (62–64). Therefore, perturbations of either glycolysis or OXPHOS compromise NK cell metabolic integrity and effector capacity. Pharmacological glycolysis inhibition with 2-DG prevents NK cell degranulation (CD107a), actin polarisation at the immune synapse, and IFN-γ production (20, 21, 65), while genetic deletion of the key glycolytic enzyme lactate dehydrogenase A (LDHA) in NK cells similarly prevents effective antiviral function and viral control *in vivo (*65), collectively demonstrating a non-redundant requirement for glycolysis in NK cell effector responses. The DC-NK cell immune synapse, especially the synaptic exocytosis (perforin/granzyme release), is also sensitive to metabolic perturbations (20, 65).

Converging pathological asthmatic processes create a nutrient- and energy-competitive environment for NK cells. Allergic inflammation induces a strong commitment to glycolysis in proliferating Th2 cells, supporting their expansion and inflammatory response (17). The combination of intense metabolic activity and the massive infiltration of eosinophils and activated DC creates substantial glucose competition at inflammatory loci (66) that is further escalated by highly glycolytic type 2 innate lymphoid cells (ILC2). ILC2 are primary sources of IL-5 and IL-13 in T2-high asthma (67), exacerbating nutrient competition and increasing NK cell metabolic pressure. Their numerical superiority and sustained glycolysis outcompete NK cells, promoting persistent nutrient deprivation and limiting NK cell effector functions. Additionally, airway remodelling and epithelial dysfunction induce tissue hypoxia, reshaping NK cell function through multiple mechanisms (10, 62–64). First, it prevents migration to viral replication sites by downregulating key chemokine receptors (CXCR3, CXCR4) and adhesion molecules (35, 36, 68). Second, while HIF-1α stabilization initially promotes glycolytic adaptation (10, 28, 64), it induces negative phenotypic alterations: the expression of activating receptors (NKG2D, NKp46, DNAM-1) is reduced while inhibitory receptors (NKG2A) increases, collectively suppressing NK cell responsiveness (35, 68). Furthermore, hypoxia arrests NK cell maturation from CD56^bright^ to the cytotoxic CD56^dim^ subset, reducing perforin/granzyme secretion despite maintained intracellular granules (36, 64). In severe asthma, chronic hypoxic exposure promotes a distinct “exhausted” NK cell phenotype characterized by PD-1 upregulation and metabolic reprogramming toward compensatory OXPHOS, resulting in dysregulated degranulation capacity even upon re-challenge under normoxic conditions (62–64). Therefore, hypoxia induces metabolic checkpoint failure by both preventing effective recruitment and limiting effector capacity, even when cytokine licensing signals are present. Mechanistically, NK cell cytotoxicity requires optimal energy supply for immune synapse formation, trafficking of lytic granules, and coordinated release of perforin and granzymes. Low-glucose conditions attenuate mTORC1 activity, reducing the translation of granzyme B and other effector proteins (65), while metabolic constraint disrupts cytoskeletal dynamics and synapse stability (41, 69), contributing unresolved inflammation.

IFN-γ production is tightly linked to glycolysis and represents the key NK cell cytokine that actively suppresses type 2(T2) immunity through multiple mechanisms: directly inhibiting Th2 cells differentiation and IL-4/IL-5/IL-13 production, suppressing activation and proliferation of ILC2, and limiting eosinophil recruitment and survival (4, 62). In asthmatic airways, IFN-γ deficiency disrupts this Th1/Th2 balance, unopposing T2 inflammation with Th2 cells expansion, ILC2-related cytokine production, and eosinophil-mediated tissue damage, reinforcing airway hyperresponsiveness (4, 62). Mechanistically, glycolysis regulates IFN-γ synthesis at multiple levels: glycolytic enzymes (GAPDH) suppress IFN-γ mRNA translation in resting NK cells, and cell activation relocates GAPDH to glycolysis metabolism, relieving mRNA for IFN-γ synthesis (4, 20, 62). Lung tissue-resident CD56^bright^CD16^low^NK (lrNK) cells exhibit specialized metabolic adaptations for survival in glucose-restricted environments (62, 70), including upregulated GLUT1 expression and inherent high glycolytic capacity to support IFN-γ production. Transcriptome analysis revealed elevated *ID3*, *IRF4*, *CXCR6*, *RGS1*, *RGS2*, and *ZNF683* expression (62, 70), supporting lrNK tissue retention, metabolic flexibility, and elevated cytokine production. The elevated glycolytic capacity and glucose dependence of lung CD56^bright^NK cells, particularly in tissue-resident subsets, enable rapid IFN-γ production upon activation through increased biosynthetic and energetic demands (62, 70). However, these NK cells display reduced IFN-γ production in response to IL-12/IL-18 or viral stimuli and compromised cytotoxicity although retaining high intracellular perforin and granzyme levels (10–12). These functional deficits are accompanied by compensatory upregulation of OXPHOS, compatible with a model of metabolic exhaustion rather than numeric deficiency (10–12) and indicating a decoupling between cytotoxic machinery and effective target lysis. Notably, while cytokine-driven IFN-γ synthesis can proceed independently of glycolytic and oxidative pathways, receptor-mediated IFN-γ production requires OXPHOS and glycolytic metabolism (70). These metabolic features, together with the high glycolytic capacity and glucose dependence of lung lrNK cells, render them highly sensitive to local glucose availability and likely to mount robust interferon responses when metabolic substrates increase during lung infections or inflammation. This specialization is supported by primary data showing that sorted human CD56^bright^ NK cells exhibit 3−fold higher glycolytic capacity (ECAR) than CD56^dim^ cells (62), and transcriptomics evidence confirms elevated glycolytic gene expression (*SLC2A1, HK2, LDHA*) in lung−resident populations (70).

In the DC–NK cell metabolic checkpoint model, lrNK cells operate in direct glucose competition with highly glycolytic lung DCs in a nutrient-deficient environment (45), resembling tumoral microenvironments (hypoxia, lactate, NK exhaustion) but with fundamental differences. For instance, asthma involves episodic pathogen clearance promoted by Th2/ILC2/eosinophils, contrasting with the persistent malignancy and tumor-associated macrophages and Tregs environment of tumors. Therefore, direct translation of tumor microenvironments, especially those related to lactate suppression (21, 40, 41) and metabolic checkpoint failure, to acute viral exacerbations in asthma requires empirical validation. Highly glycolytic DCs can deplete perisynaptic glucose at DC–NK cell interfaces, potentially starving lrNK cells even if bulk glucose appears adequate, and lactate accumulation can further suppress lrNK cell glycolysis and IFN-γ production via intracellular acidosis. This may explain why tissue-resident, memory-like NK cell subsets (e.g., CD49a^+^NKG2C^+^KIR^+^) are depleted or functionally compromised in severe asthma patients (10–12). Collectively, these data converge on a “double metabolic hit” model: glucose deprivation and nutrient competition combined with suppression of glycolytic and mitochondrial pathways by lactate and acidosis (22, 40, 41). This composite stressor constrains NK cells cytotoxicity and IFN−γ production, destabilizes Th1/Th2 balance, and contributes to the failure of innate antiviral defense observed in severe asthmatic patients.

## 5 Asthma endotypes and the DC–NK cells metabolic checkpoint

T2-high eosinophilic asthma is defined by high levels of IL-4, IL-5, and IL-13 regulated by ILC2, Th2 cells, and eosinophils, alongside elevated basal autophagy in epithelial cells, eosinophils, and DCs (17, 25). ILC2, as early responders to epithelial alarmins (IL-33, IL-25, TSLP), amplify T2 inflammation and may interact with DCs to modulate their metabolic state and cytokine production (67), though their direct impact on the DC-NK cell metabolic checkpoint remains unexplored. Autophagy in allergen-exposed DC favours highly glycolytic metabolism and Th2-polarization (17) but whether these allergen-primed DCs retain capacity for viral-induced secretory autophagy and IL-12/15 production remains unknown. Conversely, T2-low/neutrophilic asthma is associated with reduced DCs autophagy and Th17 immunity, suggesting maladapted DCs and IL−12/IL−15/IL−18 signaling for NK cell metabolic licensing. Obesity-related asthma introduces further complexity via systemic insulin resistance, hyperleptinemia, and lipid dysregulation, enforcing airway DCs into glycolytic and pro-inflammatory states and aggravating NK cell metabolic dysfunction (71), though direct evidence is scarce. Therefore, in this review, “autophagy failure” is used in an endotype− and cell−specific context to describe a potential inability of DCs to regulate basal versus stimulus-induced secretory autophagy in response to viral challenges, acknowledging that detailed human data are limited. These endotype-specific patterns of DCs metabolism, NK cell dysfunction, and metabolic checkpoint failure are summarized in **Table 1**.

**Table 1:** Endotype-specific DC–NK cell metabolic checkpoint profiles and therapeutic vulnerabilities in asthma. .

Asthma endotype	DCs metabolic profile	NK metabolic state	Primary checkpoint defect	Metabolite signature
T2-high Eosinophilic	Hyper-glycolytic (17, 28, 45); ↑ORMDL3 (73, 76); ↑Ceramides (C16:0/C18:0) (71, 77–79); ↑mTOR (26); ↓Secretory autophagy (17, 24)	Glucose-starved (66); OXPHOS compensatory upregulation (10–12); blunted IFN-γ (10–12, 62)	Cytokine axis failure (IL-12/IL-15/IL-18↓) (9, 18, 24) + ILC2 nutrient competition (67) + perisynaptic glucose depletion (66, 67)	↑Lactate (22, 23); ↓Glucose (66); ↓pH (29–31); ↑Ceramides (71, 77–79)
T2-low Neutrophilic	↓Basal autophagy (43); ↓DCs maturation/deficient tolerogenic pathways (43, 74, 81); Th17-skewed (16, 17, 43).	Metabolically preserved but deprived of IL-12/IL-18 licensing signals (5); blunted IFN-γ despite adequate glycolytic capacity (9, 62, 63).	Secretory autophagy failure (24, 43) + impaired cytokine licensing + loss of tolerogenic checkpoint (43, 74, 81)	Lactate (moderate) (22, 23); pH (variable) (29, 30, 34); ceramides altered (SPT) (71, 77).
Obesity-Related Asthma	Hyper-glycolytic, systemic insulin resistance, hyperleptinemia, lipid dysregulation, mTOR hyperactivation (23, 26).	Metabolically exhausted from dual burden: local nutrient depletion + systemic metabolic dysfunction; Blunted OXPHOS (62–64).	Convergence: systemic metabolic dysregulation (23, 25) + intrinsic checkpoint failure + chronic low-grade inflammation	↑Lactate (22, 23, 34); ↑TG (23, 71); glucose (dysregulation) (23, 71); ↑FFAs (23, 71).

Column definitions: (1) DCs Metabolic Profile: Predominant glycolytic vs. oxidative state, autophagic capacity, and upstream regulators (HIF-1α, mTOR/AMPK balance, ORMDL3–ceramide axis). (2) NK Cell Metabolic State: Glycolytic capacity, OXPHOS competence, and functional output (cytotoxicity, IFN-γ). (3) Primary Checkpoint Defect: The dominant mechanism(s) by which DCs dysfunction licenses NK cell failure (cytokine axis, exosomal signaling, nutrient competition). (4) Key Metabolite Signature: Biomarkers detectable in sputum, BALF, or serum that characterize each endotype’s metabolic perturbation. (5) Proposed Therapeutic Angle: Prioritized interventions based on the endotype-specific checkpoint defect. Abbreviations: FFAs, free fatty acids; GLUT1, glucose transporter 1; HK2, hexokinase-2; IL, interleukin; ILC2, type 2 innate lymphoid cells; mTOR, mechanistic target of rapamycin; NK, natural killer; OXPHOS, oxidative phosphorylation; SCFA, short-chain fatty acid; SERCA2, sarcoplasmic/endoplasmic reticulum calcium-ATPase 2; SPT, serine palmitoyltransferase; Th, T helper.

Immunity and tolerance are tightly controlled by DC autophagy and the mTOR/AMPK signaling axis. A key contradiction in asthma is the inconsistent description (“increased,” “decreased,” or “dysregulated) of autophagy levels for different cell types, endotypes, and functional contexts (72). As summarized in **Table 2**, structural (epithelium, smooth muscle) and T2 effector (eosinophils) cells exhibit elevated basal autophagy (39, 72, 73). Recent evidence shows increased basal autophagy in allergen-exposed DCs (17), while viral exposure triggers a distinct autophagic mechanism emphasizing cytokine production (24). Whether asthmatic DCs exhibit excessive allergen-driven basal autophagy alongside deficient viral-induced secretory autophagy remains untested in humans. This dual pattern of high basal autophagy in structural/effector compartments and deficient secretory autophagy in DCs may explain the presence of chronic inflammation (failed resolution) alongside defective viral clearance (failed activation), even though causal links remain to be fully defined. In steady-state conditions, autophagy provides immunological tolerance by removing faulty mitochondria and suppressing unwanted inflammasome activity (24, 39, 74). This continuous, low-level process is regulated by the AMPK/mTOR axis, utilizing canonical ATG proteins (26). Viral infections induce “secretory autophagy”, functional and mechanistically divergent from degradative bulk autophagic mechanisms and stimulated by mTORC1-dependent glycolysis (24). This specialized pathway hijacks key autophagy components, such as Atg5 and Beclin-1, delivering cytosolic viral antigens to endosomal TLR7/8 and promoting IL-12, IL-1β, and IL-18 secretion through autophagosome−mediated exocytosis (24). However, direct quantification of this stimulus-induced DCs secretory autophagy is currently limited to murine and *in vitro* studies (24, 39) and has not yet been analysed in airway DCs from asthmatic patients. Findings regarding DCs autophagy are context-dependent. For instance, recent research demonstrated that HDM exposure upregulated autophagy and promoted pathogenic Th2/Th17 polarization in mice (17), challenging our model. Clarifying how stimulus type (allergen vs. viral PAMPs), activation state, and specific pathways (tolerogenic vs. secretory) influence these outcomes is required for understanding human asthma. In this context, dysregulation of this “metabolic checkpoint” indicates DCs autophagic failure, locking them in a state of persistent mTOR activation (26, 48). This continuous activation is predicted to prevent tolerogenic autophagy and attenuate the acute secretory autophagic flux required for effective antiviral responses. However, current evidence for autophagy dysregulation in asthma is predominantly derived from whole-tissue analyses (39, 73, 75). For instance, bronchial biopsies demonstrate elevated LC3−II and ATG5 expression (43, 46), yet they represent accumulated signals from disrupted epithelial cells and infiltrating granulocytes, interfering with the specific metabolic status of rare airway DCs. Additional studies in asthmatic patients have confirmed intrinsic autophagy alterations in the airway epithelium (linked to ORMDL3- mediated endoplasmic reticulum stress) (73, 76) and in inflammatory cells (eosinophils and neutrophils) from sputum (39), correlating with disease severity and reflecting a upregulated basal autophagy in structural and effector cells. Mechanistic insight into DCs autophagy has largely depended on genetic models. Conditional deletion of Atg5 in CD11c^+^ cells promote neutrophilic airway inflammation and Th17 polarization (43), demonstrating that CD11c^+^−cell autophagy is required for immune tolerance. However, it also emphasizes the loss of tolerogenic autophagy rather than failure of stimulus−induced secretory autophagy during viral challenge. Experimental infection models in healthy cohorts using influenza, RSV, and rhinovirus have documented DCs activation and cytokine production (2–4, 7, 8), yet comparative analyses of autophagy flux in DCs from asthmatic versus non−asthmatic patients are lacking. Future work must bridge this gap by isolating DCs from human BALF during controlled viral challenge, followed by functional autophagy flux assays and cytokine profiling.

**Table 2:** Cell type–specific autophagy and metabolic profiles in asthma relevant to the DC–NK cell checkpoint.

Cell type	Autophagy status in asthma	Dominant metabolic phenotype	Functional consequence	Evidence level
Airway epithelium	↑Basal autophagy (ORMDL3–SERCA2–ER axis) (73, 76)	↑Glycolysis (25, 28)	Support chronic inflammation and tissue remodeling; indirectly increases nutrient demand and lactate load; Mucus secretion; Barrier dysfunction (73, 79)	Human biopsies (73, 76) and primary cell studies (43)
Airway smooth muscle	↑Basal autophagy (72, 73)	↑Glycolysis (25, 28)	Contribute to airway remodeling and hypoxia, worsening metabolic stress environment; Proliferation (25, 28)	Human and animal models (25, 72)
Eosinophils	↑Basal autophagy correlating with disease severity (22, 72)	↑Glycolysis (66)	Survival; Degranulation; Amplify T2 inflammation and local nutrient consumption (39, 66, 67)	Human sputum and BALF studies (39, 72)
Neutrophils	↑Basal autophagy in severe disease (22, 72)	↑Glycolysis (22)	Promote neutrophilic inflammation, tissue damage, and worsen glucose/lactate burden; ROS production (22, 39, 43)	Human and murine data (39, 43)
Dendritic cells	↓Basal/tolerogenic autophagy (43, 74); Secretory autophagy (deficient in asthma) (17, 24)	↑Glycolysis (HIF-1α- and mTOR-regulated); ↓Autophagic plasticity (28, 45, 96)	Defective tolerance (43, 74), reduced IL-12/IL-15/IL-18 (24), defective exosomes (91–93), perisynaptic glucose deficit → DC–NK cell checkpoint failure (27, 45)	Strong murine/*in vitro (*17, 24, 43); limited human, cell-resolved data
Plasmacytoid DC	Not characterized in human asthmatic airways; AMPK-driven catabolic reprogramming required for IFN-I production (54)	AMPK-regulated; LDHB-dependent pyruvate/lactate flux for IFN-I synthesis (59); susceptible to lactate-mediated suppression	Impaired type I IFN output in asthma via FcϵRI/IgE axis (55, 56), reduced pDC numbers (55, 56), diminished upstream priming of cDC IL-15Rα expression and direct NK IFNAR signaling (53)	Mechanistic inference from non-asthmatic contexts
NK cells	Autophagy needed for fitness (86);	Glycolysis + OXPHOS required (in asthma: metabolic exhaustion, ↑OXPHOS compensation) (20, 21, 62, 70)	Reduced cytotoxicity and IFN-γ (10, 62); failure to restrain T2 inflammation (4, 62) and clear viral infection (62–64)	Human functional studies (10); mechanistic animal work (21)
ILC2	Not established in asthma; autophagy role in ILC2 survival/proliferation unexplored	Hyper-glycolytic; sustained glucose uptake via GLUT1/mTORC1 to support IL-5/IL-13 (67)	Amplify glucose depletion; sustained T2 inflammation via IL-5/IL-13 production; ILC2–DC alarmin crosstalk (IL-33, IL-25, TSLP) may modulate DC autophagic state (67)	Mechanistic inference from Section 4

Summary of autophagy status and dominant metabolic programs across major airway and immune cell populations in asthma, and their predicted impact on the DC–NK cell metabolic checkpoint.

ORMDL3 represents the strongest and most replicated genetic association with childhood-onset asthma (17q21 locus) (13–15, 74), but evidence for its involvement in DCs autophagy in human asthma is absent (**Table 3**). Risk alleles elevate ORMDL3 expression, and preclinical studies indicate that they can promote basal autophagy via sarcoplasmic/endoplasmic reticulum Ca^2+^ ATPase via SERCA2 inhibition and endoplasmic reticulum stress (73, 76). However, ORMDL3 also modulates autophagy flux by disrupting ceramide metabolism via serine palmitoyl transferase inhibition (77, 78). For instance, pathologic ORMDL3 overexpression dysregulates ceramide homeostasis, depletes endoplasmic reticulum calcium via SERCA2 inhibition, and triggers the unfolded protein response, inducing basal autophagy, barrier dysfunction, and mucus hypersecretion in epithelial cells (73). It also promotes accumulation of ceramides (e.g. C16:0/C18:0) that can activate mTORC1 and attenuates secretory autophagy following viral challenge (79). This suggests an “autophagy imbalance” model: airway structural cells display detrimental basal autophagy and tissue remodelling, whereas immune cells, especially DCs, exhibit defective secretory autophagy, potentially favouring immunodeficiency. The ORMDL3–ceramide–autophagy axis seems further influenced by asthma endotype. T2-high asthma correlates with elevated ceramide levels and ORMDL3-regulated Th2 inflammation, whereas neutrophilic asthma may present distinct sphingolipid profiles (71). Human studies partially validate of the ORMDL3-autophagy axis in airway epithelium. Studies in primary human bronchial epithelial cells revealed that ORMDL3 overexpression promoted autophagy through SERCA2 inhibition/endoplasmic reticulum calcium depletion (73), confirming LC3-II/ATG5 expression from bronchial biopsies. Mechanistic extrapolation from epithelial studies seems plausible, but functional validation in airway DCs is required before therapeutic targeting.

**Table 3:** Methods and criteria used to assess the quality of evidence supporting each component of the proposed DC–NK cell metabolic checkpoint, and to assign validation priority. .

Checkpoint component	Human evidence	Preclinical evidence	Evidence gap	Validation priority
DCs autophagy required for IL-12/IL-15/IL-18 secretion	None	Strong (43, 44)	Human airway DCs measurement during viral infection	Priority 1
DCs autophagy deficient in asthma	None	Strong (CD11c-Atg5fl/fl) (43)	Human DC-specific autophagy status (basal vs. stimulus-induced)	Priority 1
ORMDL3 affects DCs autophagy	None	Limited (71, 73)	ORMDL3 expression-autophagy correlation in human airway DCs	Priority 1
NK cells metabolically exhausted in asthma	Strong (5, 10–12, 71)	Limited (10, 71)	Mechanistic link to DCs dysfunction	Priority 2
Lactate suppresses NK cell function	None (in asthma context)	Strong (22, 40, 41, 64, 98)	BALF lactate levels + NK cell function correlation during viral exacerbations	Priority 2
DCs IL-12/IL-15/IL-18 deficient during viral asthma	None	Moderate (5, 45)	Direct cytokine measurement from asthmatic DCs post-viral challenge	Priority 2
ILC2–DC alarmin crosstalk modulates DC autophagy and NK cell licensing	None	None (inferred from ILC2 alarmin biology and DC metabolic reprogramming (67)	Whether ILC2-derived alarmins suppress DC secretory autophagy and IL-12/IL-15 output during viral co-challenge; whether ILC2–DC crosstalk amplifies perisynaptic glucose depletion and reduces NK cell metabolic licensing	Priority 2
Hyper-glycolytic DCs create glucose-depleted microenvironment	None	Indirect (tumor models) (41, 46, 69)	Perisynaptic glucose at DC-NK cell interface	Priority 3
DCs exosomes deliver metabolic-suppressing cargo to NK cells	None	Moderate (91–93, 95)	Asthmatic DCs exosome cargo + NK cell metabolic impact	Priority 3
mTOR/AMPK axis dysregulated in asthmatic DCs	Indirect (whole tissue) (26)	Strong (17, 26, 39, 48)	DC-specific phospho-mTOR, phospho-AMPK during viral infection	Priority 3

Evidence quality was graded separately for human and murine/*in vitro* data based on study design (*in vivo* vs. ex vivo vs. cell line), relevance to asthma and viral exacerbations, reproducibility across independent studies, and mechanistic depth (pathway-level interrogation vs. descriptive association). Components were then categorized as having strong, moderate, limited, or no evidence in humans and experimental models, with explicit identification of unresolved gaps. Validation priority was ranked in three levels (Priority 1–3) according to: (i) Key mechanistic components requiring validation before therapeutic development (key mechanism vs. supportive components); (ii) potential impact of confirmation or refutation on therapeutic development, (iii) feasibility and ethical acceptability of targeted experimental approaches.

Beyond genetic influences, DCs metabolic phenotypes are dynamically regulated by the mTOR/AMPK axis. Viral activation induces an optimal window of mTORC1 activity, supporting glycolysis and protein synthesis while preserving sufficient autophagic flux (26, 61). However, chronic inflammation, metabolic competition, and hypoxia promote persistent mTORC1 activation in DCs, favouring glycolysis over autophagy (26, 61), suppressing MHC-II expression, and compromising cross-presentation (43, 44, 74). Although metabolic stress triggers AMPK, this is often insufficient to restore autophagy and anti-inflammatory responses (48) in a nutrient-deficient, cytokine-rich environment of asthmatic airways. Therapeutic agents like metformin (AMPK activator) and rapamycin (mTOR inhibitor) ameliorate allergic airway inflammation and partially restore autophagic plasticity in murine models (80, 81), but analogous mechanisms in human asthma have yet to be demonstrated. These findings suggest that checkpoint dysfunctions are subset-specific. For instance, human airway cDC1s (CD141^+^/BDCA−3^+^), optimized for cross-presentation and CD8^+^ T-cell priming, require robust OXPHOS and fatty acid oxidation to support MHC-I loading and redox balance (51, 52). Their key role in antiviral immunity involves licencing NK cells through IL-12/IL-15 (82, 83). In asthma, reduced cDC1 frequency (13–15) and potential metabolic dysregulation by chronic hypoxia and nutrient depletion may prevent licensing capacity (82, 83). DCs metabolism can further be regulated by epithelial mediators like Clara cell 10, inhibiting CD11b^+^DC activation through NF−κB suppression and preventing Th2 polarization (84). Conversely, cDC2 (CD1c^+^/BDCA−1^+^), responsible for Th2/Th17 polarization, adopt a high glycolytic, “inflammatory” phenotype (inf−cDC2s) under type-I IFN and mTOR control during viral infection (52). These inf−cDC2s elevate lactate production and pro-inflammatory cytokine secretion (52) while producing negligible IL-12 (52), potentially starving NK cells and diverting immunity from protective antiviral responses toward pathogenic inflammation. CD11b^+^CD1c^+^cDC2s also preferentially respond to allergens (HDM) and dominate T2-high asthma (13–15, 17). Whether asthmatic airways exhibit an unfavourable cDC1:cDC2 ratio promoting glycolytic, Th2-polarizing DCs over IL-12-producing, NK-licensing cDC1s during viral infections remains untested in humans.

Collectively, these data suggest that metabolic checkpoint failure in asthma results from the confluence of inherent genetic predisposition (like ORMDL3–ceramide signaling), external signaling imbalances (mTOR/AMPK), and compromised epithelial function. This locks DCs into a glycolytic state, promoting chronic Th2/Th17 inflammation and preventing NK cell function.

## 6 The metabolic checkpoint

The metabolic crosstalk between DCs and NK cells in the asthmatic lung is orchestrated through three complementary mechanistic axes, each contributing to the failure of the proposed metabolic checkpoint.

### 6.1 Axis 1: cytokine-mediated metabolic reprogramming

IL-15 is essential for survival and metabolic priming of NK cells, requiring cell-to-cell interaction with 15Rα (18, 85). IL-15/IL-15Rα interaction triggers biphasic NK cell metabolic programs, initially promoting autophagy and metabolic flexibility via the Tbkbp1–Ulk1–mTORC1 axis (86), and elevating glycolytic capacity to support effector function (20, 63). Therefore, insufficient IL-15 production or trans-presentation by asthmatic DCs is expected to compromise NK cell metabolic adaptations. IL-15 trans-presentation by DCs is mechanistically established through primary immunoprecipitation and imaging studies showing IL-15Rα:IL-15 complexes on DC membranes activating NK cells in trans (18, 85, 86) (established mechanism). Furthermore, the role of the Tbkbp1-Ulk1-mTORC1 axis in IL-15-induced autophagy is demonstrated in Tbkbp1^-/-^NK cells models (86), yet its direct involvement in asthma remains unverified. Whether DCs in asthmatic airways show reduced IL-15 trans-presentation during viral infections is hypothetical, lacking direct human measurements of IL-15Rα^+^DC frequency or IL-15 bioavailability in BALF during exacerbations. Upstream of this axis, type I IFNs from pDC upregulate IL-15Rα on cDCs, allowing IL-15 trans-presentation (18, 51, 52). Therefore, the pDC IFN-I deficiency observed in asthmatic airways (55, 60) may reduce cDC1s IL-15 trans-presentation to NK cells, representing an additional, upstream contributor to Axis 1 failure that warrants experimental quantification.

IL-12 is a classical Th1-polarizing cytokine induced by TLR stimulation and facilitated through autophagy (9). It activates mTOR in NK cells, promoting IFN-γ production and glycolysis (9, 63). In asthma, deficient autophagy in DCs reduces IL-12 output (5), limiting NK cell activation and metabolic priming. Furthermore, IL-12 can amplify cell stimulation and NK cell effector function in combination with IL-18, a powerful inducer of IFN-γ in NK cells through MyD88−dependent pathways (87, 88). Rather depending on mTOR signaling, IL-18 maturation requires inflammasome activation and its secretion is facilitated by autophagy, indicating attenuated IL−18 secretion in asthma. However, disruption of key autophagy molecules (ATG16L1 or ATG7) in myeloid cells elevates IL−18 output (89) and allergen-induced autophagy promotes IL-18 release from airway epithelium (90), indicating that intact autophagy stimulates its production. These findings support autophagy not as an absolute prerequisite but as a context−dependent regulator for IL−18 production, further modulating NK cell function. Collectively, we hypothesize that compromised IL−12, IL−15, and autophagy−regulated IL−18 signalling in asthmatic DCs may suppress NK−cell metabolic programming, though direct human evidence during viral exacerbations is currently lacking. Measurements in stable asthma or allergen models cannot be extrapolated to viral contexts due to distinct pattern recognition receptor signaling.

### 6.2 Axis 2: exosome-mediated metabolic reprogramming

Exosomes are nanoscale vesicles containing proteins, lipids, mRNAs, and miRNAs facilitating intercellular communication (91, 92). DC-derived exosomes (DEX) actively support NK cell function, containing IL-15/IL-15R and NKG2D ligands (MICA/B, RAE-1γ) (91, 92). Conversely, tumor-derived exosomes (TEX) suppress NK cell activity through multiple mechanisms: leveraging NKG2D ligands (MICA/B, ULBP1-6) that chronically engage and progressively downregulate NKG2D, and delivering miRNAs (miR-92b and miR-23a) and TGF-β that inhibits IFN-γ production and prevents NK cell degranulation/infiltration. However, a direct mechanistic showing TEX components reprogramming NK cell glycolytic metabolism is currently lacking. Asthmatic airways also secrete exosomes with immune-metabolic miRNA, including miR−155 and miR−21 (93, 94), promoting Th2 polarization and OXPHOS suppression in recipient cells (94, 95). Additionally, DEX containing miR−493−5p targets IRF-4 and FOXO1 pathways, modulating inflammation (93) and potentially altering NK cell function. Direct validation of these cargo-transfer mechanisms and their impact on NK cell metabolism in asthmatic conditions is current absent. This axis represents the weakest component of the proposed checkpoint, requiring proteomic and lipidomic characterization DEX during viral infection. TEX mechanisms may also not directly translate to asthma due to fundamental differences in cellular sources (tumor cells vs. inflamed epithelium/immune cells), cargo composition (tumor-specific antigens vs. allergen/viral PAMPs), and functional goals (tumor immune evasion vs. pathogen clearance failure) (91–93, 95). Asthma-specific exosome profiling during viral exacerbations is essential before extrapolating cancer immunometabolism findings.

### 6.3 Axis 3: nutrient competition

**Figure 1:**
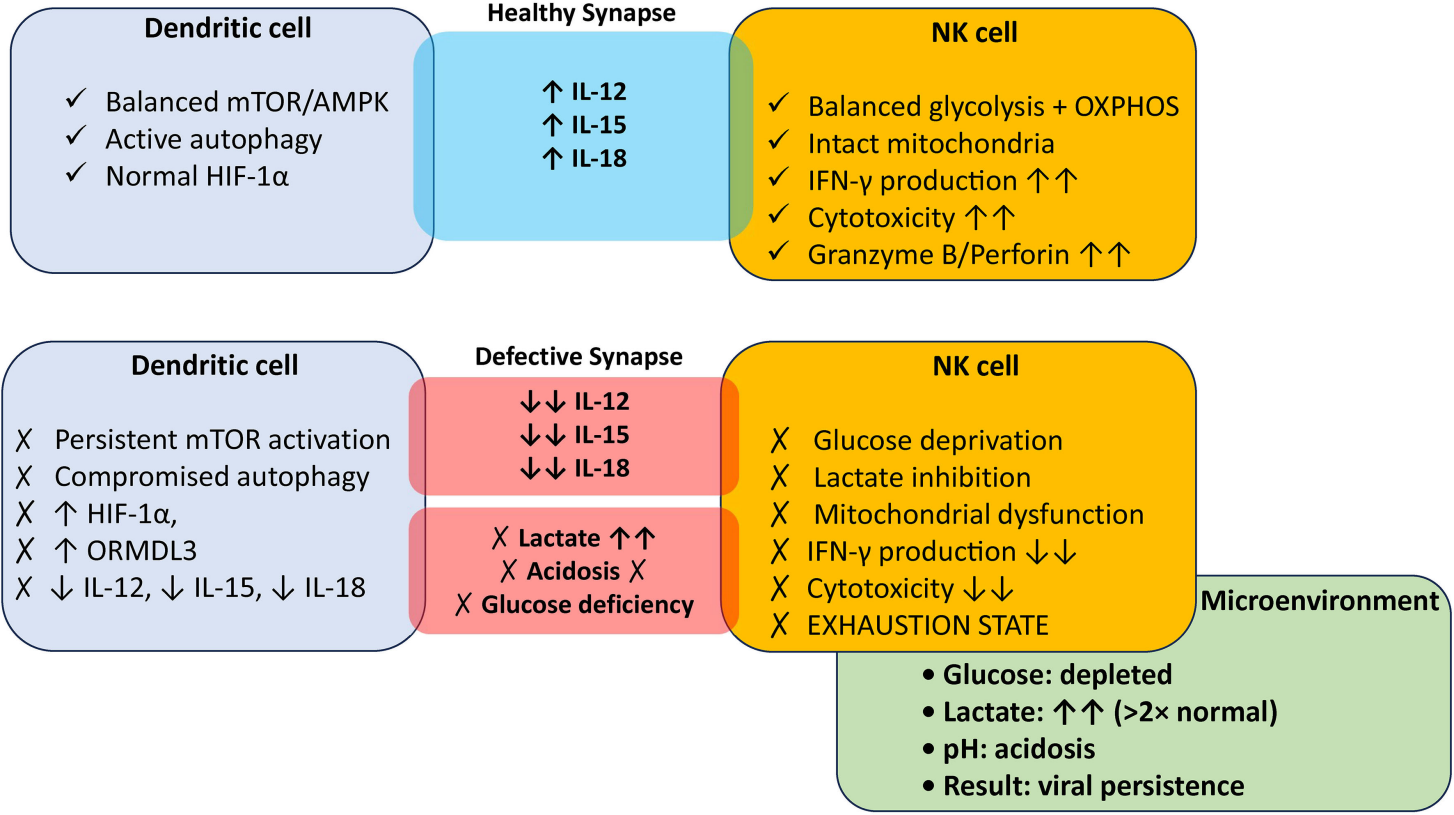
The DC–NK cell metabolic checkpoint model explains divergent immune outcomes in health versus asthma. **(A)** Healthy lung: Dendritic cells maintain balanced mTOR/AMPK signaling and autophagic plasticity, enabling robust IL-12, IL-15, and IL-18 secretion. These cytokines, acting on NK cells in a glucose-rich, neutral pH microenvironment, promote coordinated glycolysis and OXPHOS upregulation, resulting in high IFN-γ production and effective cytotoxicity against virally infected cells. **(B)** Asthmatic lung: Chronic HIF-1α stabilization and mTOR hyperactivation impair DCs autophagic plasticity, dramatically reducing IL-12/IL-15/IL-18 output. Simultaneously, hyper-glycolytic DCs create a perisynaptic glucose sink while lactate accumulates to pathologic concentrations, acidifying the microenvironment to pH <6.8. NK cells experience a “triple metabolic hit”: deficient cytokine licensing, glucose starvation, and lactate-mediated glycolytic inhibition. The result is functional NK cell anergy with impaired IFN-γ production and cytotoxicity, permitting viral persistence and exacerbation-prone airway inflammation. Green checkmarks (✓) indicate metabolic competence; red X marks (✗) indicate dysfunction; double arrows (↑↑/↓↓) denote magnitude of change.

The third mechanism is direct competition with highly glycolytic immune cells consuming available nutrients, with DCs especially sequestering glucose from NK cells at the immune synapse. Although airway glucose often increases during asthmatic inflammation due to plasma leakage, direct measurements during acute viral exacerbations are missing. In asthmatic airways, hypoxia stabilizes HIF-1α, inducing upregulation of GLUT1 and glycolytic enzymes in infiltrating cells (96, 97), enforcing glucose uptake and generating lactate as glycolysis byproduct. Lactate accumulates in airways, exceeding healthy controls levels (22, 23, 29–31), decreasing interstitial pH, and promoting intracellular acidification that suppress NK cell function (10, 64). This creates a “double hit”: glucose sequestration by hyper-glycolytic cells depletes energy sources and lactate accumulation suppress NK cell function (34, 40, 41, 64, 98). Whether DC-specific glycolysis substantially contributes to airway lactate accumulation remains unknown, and alternative sources include β-agonists (type B lactic acidosis), respiratory muscle work and fatigue during bronchoconstriction, and bulk inflammatory cell metabolism (22, 34). The entire nutrient competition hypothesis rests on three unproven assumptions requiring *in vivo* validation: 1) Highly glycolytic DCs deplete glucose at immune synapses, creating local depletion zones (never measured *in vivo*); 2) This depletion suppresses NK cell metabolic function (extrapolated from *in vitro* 2-DG studies (20, 21) and not demonstrated at physiological DC: NK cell ratios); and 3) DC-specific glycolysis substantially contributes to total airway lactate [unquantified, as airway lactate derives from multiple sources (22, 34)] (**Figure 1**). These three proposed axes, cytokine-mediated licensing, exosome transfer, and nutrient competition, vary substantially in evidence quality (**Table 3**), with the first axis having strongest support and the second requiring validation. Therefore, primary studies using isotope tracing (^13^C-glucose) tracking DC-specific contributions, combined with microelectrode pH measurements at DC-NK cell interfaces, are required to validate this axis.

## 7 Therapeutic opportunities: targeting the metabolic checkpoint

Restoring the DC–NK cell metabolic checkpoint offers multiple therapeutic avenues, each with distinct mechanistic rationales, evidence bases, and safety considerations. If the DC-NK cell metabolic checkpoint validates in human studies, multiple therapeutic avenues deserve investigation. However, metabolic interventions (metformin, rapamycin) rely on the assumption that immune cell glycolysis, particularly DCs, meaningfully contributes to airway lactate accumulation and NK cell dysfunction. Given that β-agonist therapy and respiratory muscle work are established lactate sources (34), quantifying the contribution of infiltrating immune cells is a prerequisite to rational therapeutic development in asthma.

Neuromodulation, including electroacupuncture and vagal nerve stimulation, is emerging as a therapeutic strategy to manage lung immunity via the autonomic nervous system (99). Preclinical asthma models demonstrate that acupuncture stimulation at GV14/BL12/BL13 reduces lung CD11b^+^ DC frequency, downregulates CD86/OX40L, and decreases CCL17/CCL22 expression, alongside attenuation of Th2 airway inflammation and lung function improvement (100, 101). However, direct evidence that these approaches reprogram DCs or NK cell metabolism in asthmatic patients remains absent. Safety profiles appear acceptable in controlled settings, but procedure-related complications, device tolerability, and placebo responses warrant careful evaluation. Rigorous sham-controlled trials with mechanistic endpoints, including DCs activation markers, NK cell function assays, and metabolomic profiling, are needed to identify engagement of DC–NK cell checkpoint through neuromodulation.

Metformin is a biguanide that activates AMPK and inhibits mTOR (102), potentially modulating DCs autophagy (103) and T2 inflammation (48, 63, 80, 81). Evidence supporting metformin in asthma comes primarily from murine asthma models (95) and observational data in asthmatic patients with diabetes (104). However, the therapeutic goal is ambiguous because interventions can increase autophagy (supporting viral-induced secretory autophagy) or decrease it [suppressing allergen-driven DCs maturation (17)]. Additionally, if viral-induced secretory autophagy is already deficient in asthma, further suppression could paradoxically worsen antiviral immunity. This would reduce pathogenic allergen-induced autophagy while preserving antiviral secretory autophagy, requiring careful therapeutic windows. This contradiction must be resolved through human DCs functional studies before clinical trials. Safety considerations include the risk of lactic acidosis and potential interactions with anti-asthmatic drugs (105). Future asthma trials will need careful exclusion criteria, close metabolic monitoring, and stratification by baseline metabolic status to exclude these risks. Systemic mTOR inhibition also carries immunosuppression concerns (61). Therefore, trials would likely require inhaled or locally targeted formulations, conservative dosing, infection-related endpoints, and safety stopping rules in infection−prone asthmatic patients.

Short-chain fatty acids (acetate, propionate, and butyrate) are key metabolites produced by gut fermentation of dietary fibers, regulating systemic immunity via G protein-coupled receptors and histone deacetylase inhibition (106). They reach the lungs via circulation and mechanistic data strongly support their beneficial effect on DCs autophagy and airway hyperresponsiveness (106), but human evidence remains limited. Gastrointestinal intolerance, inter-individual microbiome variability, and standardization challenges require well-controlled trials incorporating metabolomic readouts, microbiome profiling, and clearly defined clinical endpoints identifying true checkpoint modulation from nonspecific nutritional effects.

Correcting airways acidity is a viable strategy to restore NK cell function. Nebulization with sodium bicarbonate significantly increases sputum pH and improves lung function in asthmatic patients (107). Current evidence is supported by physiologic studies showing pH-dependent immune effects, with minimal large-scale clinical trials (34, 40, 41, 107). Potential risks include bronchial irritation, altered mucus properties, and electrolyte disturbances in renal or cardiac patients. Early-phase trials require cautious dose-escalation, spirometric monitoring, and detailed airway pH with metabolite profiling.

Current asthma biologics targeting T2 pathways (anti-IL-5, anti-IL-4Rα, anti-IL-33, anti-TSLP) effectively reduce eosinophilic inflammation but show variable impact on viral exacerbation rates (108–110), reflecting their divergent effects on DC-NK cell metabolic checkpoint. These agents could theoretically decrease nutrient competition and lactate accumulation by reducing ILC2 and eosinophil glycolytic activity, potentially improving the metabolic microenvironment for NK cells (108–110). However, T2 suppression without restoring DC autophagy and IL-12/IL-15 production may fail to reactivate NK cell metabolic licensing (110, 111). Furthermore, some biologics may inadvertently inhibit DC function. For instance, TSLP blockade could affect DC maturation and migration (112). Further combination of T2-targeted biologics with metabolic checkpoint modulators (metformin, pH correction) aimed to restore antiviral immunity represents a key therapeutic question requiring clinical investigation. Biomarker studies correlating NK cell recovery with reduced viral exacerbation in biologic-treated patients would clarify whether metabolic checkpoint restoration has clinical benefits (108–110, 112).

Before initiating therapeutic any trials targeting the metabolic checkpoint, human validation studies are essential. Specifically: (1) controlled viral challenge studies with simultaneous DCs and NK cell isolation from BALF to quantify autophagy, cytokine production, and metabolic profiles; (2) isotope tracing to determine DC-specific contributions to airway lactate; (3) perisynaptic metabolite measurements at DC-NK cell interfaces; and (4) genotype-phenotype correlations (e.g., ORMDL3 variants vs. DCs autophagy). Given the 95% failure rate of preclinical-supported interventions (113), this stepwise approach minimizes risk and resource expenditure.

## 8 Critical research gaps and future directions

Current evidence for defects in DCs autophagy derives from preclinical models (24, 80, 81, 114, 115), which may not recapitulate human airway DCs biology. Furthermore, a recent murine study suggests allergen exposure increases DCs autophagy (17), though viral and allergen triggers may induce opposing effects. Additionally, most human data derive from stable patients or allergen-challenge models rather than active viral exacerbations, limiting direct validation of the DC–NK cell metabolic checkpoint hypothesis.

Elevated lactate levels are also observed in stable asthma (22, 23). However, interpreting lactate levels during acute exacerbations is challenging. For instance, high-dose bronchodilators (albuterol/salbutamol) induce type B lactic acidosis via β_2_-adrenergic stimulation and respiratory muscle work. Whether hyper-glycolytic airway immune cells contribute to this acidosis remain unverified. Direct measurement of glucose and lactate in the BALF of asthmatic patients during HRV, influenza, or RSV exacerbations would validate metabolite dynamics, identify lactate inhibitory concentrations, and correlate metabolite levels with NK cell function, IFN-γ production, and viral clearance.

Whether asthmatic DCs exhibit a suppressed (T2-high phenotype) or exacerbated (neutrophilic) autophagy (24, 74, 116) remains unresolved. Single-cell sequencing and immunofluorescence of bronchial biopsies for autophagy markers (LC3, p62, phospho-ULK1) would differentiate asthma endotypes and characterize mechanistic contributions (48, 116). Characterizing NK cell exhaustion in humans demands direct measurement of extracellular acidification and oxygen consumption rates in BALF, with differentiation between asthma endotypes. Serial BALF isolation of DCs and NK cells during controlled HRV challenges would establish the precise timeline of metabolic checkpoint failure and clarify whether hypoxia-induced NK cell phenotypic changes (altered receptor expression, maturation arrest) are reversible under normoxic rescue or standard oxygen therapy. The relative contribution of impaired NK cell recruitment (due to low pO_2_) versus intrinsic metabolic dysfunction, and the endotype-specific association between tissue pO_2_ and NK cell deficits (e.g., CD57, KIR), remain key priorities. The role of ILC2s within the DC-NK cell metabolic checkpoint also requires investigation. Highly glycolytic ILC2s are primary cytokine producers in T2-high asthma (67) and may influence DC autophagic plasticity through epithelial alarmin-mediated pathways or compete directly with NK cells for perisynaptic nutrients. Whether ILC2-DC metabolic crosstalk inhibits NK cell licencing during viral infections remains unexplored. In obesity-associated and severe asthma, direct human evidence that DCs autophagy and metabolism causally determine NK cell metabolic competence is absent. How T2-targeted biologics modulate DC-NK cell metabolic checkpoint during viral infections is equally unknown. Longitudinal studies measuring DC autophagy markers, cytokine production capacity, and NK cell metabolic profiles before and after biologic therapy, particularly during viral challenges, would determine whether suppressing T2 inflammation alone restores checkpoint function or requires complementary metabolic interventions. The proposed DC-NK cell checkpoint shares features with tumor immunology, including lactate-mediated NK cell suppression (40, 41), metabolic starvation (69), and exosomal reprogramming (91–93, 95). However, direct extrapolation is unwanted: tumor microenvironments impose constitutive immune suppression, whereas asthma involves episodic antiviral failure; tumor NK cells experience chronic antigen-induced exhaustion, while asthmatic NK cells face acute viral insults superimposed by chronic allergic inflammation. Direct validation in human asthma (measuring airway lactate during viral exacerbations and testing whether lactate neutralization rescues NK cell competence) is required before applying tumor-derived mechanistic models.

The metabolic regulation of pDCs in the asthmatic lung represents a distinct and currently uncharacterized research gap with direct relevance to the proposed checkpoint. Although their numbers and IFN-α output capacity are reduced in asthma (55, 56, 60) and LDHB-dependent metabolic capacity controls IFN-I production in viral contexts (59), no study has profiled autophagy flux, AMPK/mTOR activity, nor LDHB expression in pDCs isolated from asthmatic patients during active viral exacerbation. Given that type I IFNs promote IL-15 trans-presentation on cDCs (58, 85), rendering pDC metabolic dysfunction directly upstream of cDC-NK cell checkpoint failure should be prioritized in future human studies.

An immediate, tractable step is the reanalysis of publicy available datasets. scRNAseq datasets comparing mild vs. severe asthma, stable vs. exacerbation states, or pre/post-biologics, including GSE63142/GSE97668 (117), GSE147878 (118), and human lung cell atlases (119, 120), should be interrogated for DC subset ratios (cDC1/cDC2/pDC), metabolic signatures (GLUT1, HK2, LDHA, ATG5, LC3B, BECN1), and NK cell exhaustion and metabolic markers (CD57, KIR, NKG2D, NKG2A, SLC2A1, HIF1A), alongside correlations between DC glycolysis and NK cell function during viral events. Metabolomics datasets from sputum/BALF or documented exacerbation histories (23) should be reanalysed to link lactate levels, stratified by disease severity and β-agonist use, with viral infection rates. These *in silico* analyses offer immediate preliminary support for the model and define experimental priorities for prospective validation, as summarized in **Table 4**.

**Table 4:** Evidence quality assessment.

Claim	Primary human evidence	Primary experimental evidence	Status
NK cells exhibit reduced IFN-γ and cytotoxicity in asthma	Flow cytometry, ECAR/OCR (10);	✓ Direct measurement	ESTABLISHED
Airway lactate is elevated in asthma	BALF LC-MS (22); Sputum NMR (23)	✓ Metabolomics	ESTABLISHED
Glycolysis is required for NK cell cytotoxicity	Human NK cell with 2-DG (65); LDHA^-/-^ NK (21)	✓ Genetic/pharmacologic	ESTABLISHED
DCs require autophagy for IL-12 secretion	None in human asthma	Murine ATG5^fl/fl^CD11c-Cre (43); RSV-infected DC (24)	MECHANISTIC
Asthmatic DCs have deficient *viral-induced* secretory autophagy	None	Murine HDM model (shows *allergen* increases autophagy) (17)	HYPOTHETICAL
DCs deplete perisynaptic glucose from NK cells	None	None (extrapolated from tumor studies) (27)	HYPOTHETICAL
DC-derived exosomes reprogram NK cell metabolism in asthma	None	Healthy DC→NK (92); TEX studies (extrapolated from tumor studies) (91)	HYPOTHETICAL

Strong primary human evidence: 1) NK cell functional deficits in severe asthma: degranulation assays and IFN-γ; 2) Elevated airway lactate: LC-MS in BALF; 3) mTOR activation in exacerbations: phospho-S6/4E-BP1 from bronchial biopsies. Strong primary murine evidence: 1) ATG5-dependent DC IL-12 secretion: ATG5^fl/fl^CD11c-Cre mice; 2) Autophagy-mediated IL-12/IL-18 release: autophagy flux assays and cytokine assessemnt. Hypothetical extrapolation without primary human evidence: 1) Deficient secretory autophagy in asthmatic DCs during viral challenge: requires isolation of human airway DCs during HRV exacerbations with LC3 flux assays; 2) Perisynaptic glucose depletion by DCs: requires ^13^C-glucose tracing and DC-specific glycolysis quantification; 3) Exosome-mediated metabolic reprogramming in asthma: requires proteomic characterization of asthma-derived exosomes during viral infection. ✓ = Primary data available; ESTABLISHED = Replicated primary human studies; MECHANISTIC = Proven in model systems; HYPOTHETICAL = Logical integration without direct evidence. .

## 9 Conclusions

Asthma exacerbations remain a major unmet clinical need, and accumulating data indicate that dysregulated immunometabolism is central to this vulnerability. This review synthesizes evidence supporting the hypothesis that DCs and NK cells form a metabolic checkpoint in the asthmatic lung, in which DCs autophagic plasticity, mTOR/AMPK signaling, and nutrient handling collectively license (or constrain) NK cell antiviral competence. Across endotypes, a pattern emerges of hyper-glycolytic, ORMDL3- and HIF−1α–regulated DCs, defective cytokine and exosomal signaling (IL−12/IL−15/IL−18, miRNAs), and intense local glucose competition that together render NK cells metabolically “starved” and functionally impaired. This model remains partly inferential, particularly regarding DCs secretory autophagy and perisynaptic nutrient flux in human asthma, but it generates clear, testable hypotheses and emphasizes specific experimental priorities, including DC-specific autophagy measurements, airway metabolomics during viral challenge, and *in situ* analysis of DC–NK cell synapses. Therapeutically, targeting this checkpoint through metabolic reprogramming (e.g. AMPK activation, mTOR modulation, SCFA pathways), modulation of airway pH and nutrient availability, and neuromodulatory approaches offers a conceptual shift from broadly suppressing inflammation to restoring antiviral metabolic competence. Validating and refining this framework in human systems will be critical for developing next-generation strategies to prevent viral-triggered asthma exacerbations.

## References

[1] GBD 2015 Chronic Respiratory Disease Collaborators . Global, regional, and national deaths, prevalence, disability-adjusted life years, and years lived with disability for chronic obstructive pulmonary disease and asthma, 1990-2015: A systematic analysis for the global burden of disease study 2015. Lancet Respir Med. (2017) 5:691–706. doi: 10.1016/s2213-2600(17)30293-x. PMID: 28822787 PMC5573769

[2] Johnston SL Pattemore PK Sanderson G Smith S Lampe F Josephs L . Community study of role of viral infections in exacerbations of asthma in 9–11 year old children. Bmj. (1995) 310:1225–9. doi: 10.1136/bmj.310.6989.1225. PMID: 7767192 PMC2549614

[3] Wark PA Johnston SL Bucchieri F Powell R Puddicombe S Laza-Stanca V . Asthmatic bronchial epithelial cells have a deficient innate immune response to infection with rhinovirus. J Exp Med. (2005) 201:937–47. doi: 10.1084/jem.20041901. PMID: 15781584 PMC2213100

[4] Contoli M Message SD Laza-Stanca V Edwards MR Wark PA Bartlett NW . Role of deficient type iii interferon-lambda production in asthma exacerbations. Nat Med. (2006) 12:1023–6. doi: 10.1038/nm1462. PMID: 16906156

[5] Singanayagam A Glanville N Girkin JL Ching YM Marcellini A Porter JD . Corticosteroid suppression of antiviral immunity increases bacterial loads and mucus production in copd exacerbations. Nat Commun. (2018) 9:2229. doi: 10.1038/s41467-018-04574-1. PMID: 29884817 PMC5993715

[6] Bartlett NW Walton RP Edwards MR Aniscenko J Caramori G Zhu J . Mouse models of rhinovirus-induced disease and exacerbation of allergic airway inflammation. Nat Med. (2008) 14:199–204. doi: 10.1038/nm1713. PMID: 18246079 PMC3384678

[7] Jackson DJ Gangnon RE Evans MD Roberg KA Anderson EL Pappas TE . Wheezing rhinovirus illnesses in early life predict asthma development in high-risk children. Am J Respir Crit Care Med. (2008) 178:667–72. doi: 10.1164/rccm.200802-309OC. PMID: 18565953 PMC2556448

[8] Stein RT Sherrill D Morgan WJ Holberg CJ Halonen M Taussig LM . Respiratory syncytial virus in early life and risk of wheeze and allergy by age 13 years. Lancet (London England). (1999) 354:541–5. doi: 10.1016/s0140-6736(98)10321-5. PMID: 10470697

[9] Ferlazzo G Pack M Thomas D Paludan C Schmid D Strowig T . Distinct roles of il-12 and il-15 in human natural killer cell activation by dendritic cells from secondary lymphoid organs. PNAS. (2004) 101:16606–11. doi: 10.1073/pnas.0407522101. PMID: 15536127 PMC534504

[10] Wu H Bi J Wu G Zheng C Lu Z Cui L . Impaired cytolytic activity of asthma-associated natural killer cells is linked to dysregulated transcriptional program in energy metabolism. Mol Immunol. (2018) 101:514–20. doi: 10.1016/j.molimm.2018.08.015. PMID: 30145544

[11] Haworth O Cernadas M Levy BD . Nk cells are effectors for resolvin e1 in the timely resolution of allergic airway inflammation. J Immunol (Baltimore Md: 1950). (2011) 186:6129–35. doi: 10.4049/jimmunol.1004007. PMID: 21515793 PMC3823382

[12] Barnig C Cernadas M Dutile S Liu X Perrella MA Kazani S . Lipoxin a4 regulates natural killer cell and type 2 innate lymphoid cell activation in asthma. Sci Transl Med. (2013) 5:174ra26. doi: 10.1126/scitranslmed.3004812. PMID: 23447017 PMC3823369

[13] Cameron A Dhariwal J Upton N Ranz Jimenez I Paulsen M Wong E . Type i conventional dendritic cells relate to disease severity in virus-induced asthma exacerbations. Clin Exp Allergy: J Br Soc For Allergy Clin Immunol. (2022) 52:550–60. doi: 10.1111/cea.14116. PMID: 35212067 PMC9310571

[14] Lambrecht BN De Veerman M Coyle AJ Gutierrez-Ramos JC Thielemans K Pauwels RA . Myeloid dendritic cells induce th2 responses to inhaled antigen, leading to eosinophilic airway inflammation. J Clin Invest. (2000) 106:551–9. doi: 10.1172/jci8107. PMID: 10953030 PMC380243

[15] de Heer HJ Hammad H Soullié T Hijdra D Vos N Willart MA . Essential role of lung plasmacytoid dendritic cells in preventing asthmatic reactions to harmless inhaled antigen. J Exp Med. (2004) 200:89–98. doi: 10.1084/jem.20040035. PMID: 15238608 PMC2213319

[16] Shi LZ Wang R Huang G Vogel P Neale G Green DR . Hif1alpha-dependent glycolytic pathway orchestrates a metabolic checkpoint for the differentiation of th17 and treg cells. J Exp Med. (2011) 208:1367–76. doi: 10.1084/jem.20110278. PMID: 21708926 PMC3135370

[17] Li Y Zhou M Ren Y Li Y Xiang J Deng F . Allergen-induced dendritic cell autophagy drives immune imbalance in asthma. Int Immunopharmacol. (2026) 168:115897. doi: 10.1016/j.intimp.2025.115897. PMID: 41297347

[18] Lucas M Schachterle W Oberle K Aichele P Diefenbach A . Dendritic cells prime natural killer cells by trans-presenting interleukin 15. Immunity. (2007) 26:503–17. doi: 10.1016/j.immuni.2007.03.006. PMID: 17398124 PMC2084390

[19] Littwitz-Salomon E Moreira D Frost JN Choi C Liou KT Ahern DK . Metabolic requirements of nk cells during the acute response against retroviral infection. Nat Commun. (2021) 12:5376. doi: 10.1038/s41467-021-25715-z. PMID: 34508086 PMC8433386

[20] Mah AY Rashidi A Keppel MP Saucier N Moore EK Alinger JB . Glycolytic requirement for nk cell cytotoxicity and cytomegalovirus control. JCI Insight. (2017) 2:e95128. doi: 10.1172/jci.insight.95128. PMID: 29212951 PMC5752285

[21] Sheppard S Santosa EK Lau CM Violante S Giovanelli P Kim H . Lactate dehydrogenase a-dependent aerobic glycolysis promotes natural killer cell anti-viral and anti-tumor function. Cell Rep. (2021) 35. doi: 10.1016/j.celrep.2021.109210. PMID: 34077737 PMC8221253

[22] Ostroukhova M Goplen N Karim MZ Michalec L Guo L Liang Q . The role of low-level lactate production in airway inflammation in asthma. Am J Physiol Lung Cell Mol Physiol. (2012) 302:L300–7. doi: 10.1152/ajplung.00221.2011. PMID: 22080752 PMC3289274

[23] Ho WE Xu YJ Xu F Cheng C Peh HY Tannenbaum SR . Metabolomics reveals altered metabolic pathways in experimental asthma. Am J Respir Cell Mol Biol. (2013) 48:204–11. doi: 10.1165/rcmb.2012-0246OC. PMID: 23144334 PMC5455591

[24] Morris S Swanson MS Lieberman A Reed M Yue Z Lindell DM . Autophagy-mediated dendritic cell activation is essential for innate cytokine production and apc function with respiratory syncytial virus responses. J Immunol (Baltimore Md: 1950). (2011) 187:3953–61. doi: 10.4049/jimmunol.1100524. PMID: 21911604 PMC3186849

[25] Baay-Guzman GJ Bebenek IG Zeidler M Hernandez-Pando R Vega MI Garcia-Zepeda EA . Hif-1 expression is associated with ccl2 chemokine expression in airway inflammatory cells: Implications in allergic airway inflammation. Respir Res. (2012) 13:60. doi: 10.1186/1465-9921-13-60. PMID: 22823210 PMC3439306

[26] Zhang Y Jing Y Qiao J Luan B Wang X Wang L . Activation of the mtor signaling pathway is required for asthma onset. Sci Rep. (2017) 7:4532. doi: 10.1038/s41598-017-04826-y. PMID: 28674387 PMC5495772

[27] Reinfeld BI Madden MZ . Cell-programmed nutrient partitioning in the tumour microenvironment. (2021) 593:282–8. doi: 10.1038/s41586-021-03442-1, PMID: 33828302 PMC8122068

[28] Crotty Alexander LE Akong-Moore K Feldstein S Johansson P Nguyen A McEachern EK . Myeloid cell hif-1α regulates asthma airway resistance and eosinophil function. J Mol Med (Berlin Germany). (2013) 91:637–44. doi: 10.1007/s00109-012-0986-9. PMID: 23250618 PMC3646920

[29] Hunt JF Fang K Malik R Snyder A Malhotra N Platts-Mills TA . Endogenous airway acidification. Implications for asthma pathophysiology. Am J Respir Crit Care Med. (2000) 161:694–9. doi: 10.1164/ajrccm.161.3.9911005. PMID: 10712309

[30] Kostikas K Papatheodorou G Ganas K Psathakis K Panagou P Loukides S . Ph in expired breath condensate of patients with inflammatory airway diseases. Am J Respir Crit Care Med. (2002) 165:1364–70. doi: 10.1164/rccm.200111-068OC. PMID: 12016097

[31] Elhefny A Mourad S Morsi TS Kamel MA Mahmoud HM . Exhaled breath condensate nitric oxide end products and ph in controlled asthma. Egyptian J Chest Dis Tuberc. (2012) 61:247–56. doi: 10.1016/j.ejcdt.2012.08.007. PMID: 41865745

[32] Brown RA Spina D Page CP . Adenosine receptors and asthma. Br J Pharmacol. (2008) 153 Suppl 1:S446–56. doi: 10.1038/bjp.2008.22. PMID: 18311158 PMC2268070

[33] Kool M Willart MA van Nimwegen M Bergen I Pouliot P Virchow JC . An unexpected role for uric acid as an inducer of t helper 2 cell immunity to inhaled antigens and inflammatory mediator of allergic asthma. Immunity. (2011) 34:527–40. doi: 10.1016/j.immuni.2011.03.015. PMID: 21474346

[34] Mountain RD Heffner JE Brackett NC Sahn SA . Acid-base disturbances in acute asthma. Chest. (1990) 98:651–5. doi: 10.1378/chest.98.3.651. PMID: 2118447

[35] Parodi M Raggi F Cangelosi D Manzini C Balsamo M Blengio F . Hypoxia modifies the transcriptome of human nk cells, modulates their immunoregulatory profile, and influences nk cell subset migration. Front Immunol. (2018) 9:2358. doi: 10.3389/fimmu.2018.02358. PMID: 30459756 PMC6232835

[36] Balsamo M Manzini C Pietra G Raggi F Blengio F Mingari MC . Hypoxia downregulates the expression of activating receptors involved in nk-cell-mediated target cell killing without affecting adcc. Eur J Immunol. (2013) 43:2756–64. doi: 10.1002/eji.201343448. PMID: 23913266

[37] Liang X Arullampalam P Yang Z Ming XF . Hypoxia enhances endothelial intercellular adhesion molecule 1 protein level through upregulation of arginase type ii and mitochondrial oxidative stress. Front Physiol. (2019) 10:1003. doi: 10.3389/fphys.2019.01003. PMID: 31474872 PMC6702258

[38] Schioppa T Uranchimeg B Saccani A Biswas SK Doni A Rapisarda A . Regulation of the chemokine receptor cxcr4 by hypoxia. J Exp Med. (2003) 198:1391–402. doi: 10.1084/jem.20030267. PMID: 14597738 PMC2194248

[39] Ban GY Pham DL Trinh TH Lee SI Suh DH Yang EM . Autophagy mechanisms in sputum and peripheral blood cells of patients with severe asthma: A new therapeutic target. Clin Exp Allergy: J Br Soc For Allergy Clin Immunol. (2016) 46:48–59. doi: 10.1111/cea.12585. PMID: 26112695

[40] Brand A Singer K Koehl GE Kolitzus M Schoenhammer G Thiel A . Ldha-associated lactic acid production blunts tumor immunosurveillance by t and nk cells. Cell Metab. (2016) 24:657–71. doi: 10.1016/j.cmet.2016.08.011. PMID: 27641098

[41] Fischer K Hoffmann P Voelkl S Meidenbauer N Ammer J Edinger M . Inhibitory effect of tumor cell-derived lactic acid on human t cells. Blood. (2007) 109:3812–9. doi: 10.1182/blood-2006-07-035972. PMID: 17255361

[42] Dubey N Woodson R Hendrix SV Rosen AL Kinsella RL McKee SR . Autophagy functions in lung macrophages and dendritic cells to regulate allergen-dependent inflammatory responses. bioRxiv: Preprint Server For Biol. (2025). doi: 10.1101/2023.03.16.533006. PMID: 40799574 PMC12340803

[43] Lee HK Mattei LM Steinberg BE Alberts P Lee YH Chervonsky A . *In vivo* requirement for atg5 in antigen presentation by dendritic cells. Immunity. (2010) 32:227–39. doi: 10.1016/j.immuni.2009.12.006. PMID: 20171125 PMC2996467

[44] Schmid D Pypaert M Münz C . Antigen-loading compartments for major histocompatibility complex class ii molecules continuously receive input from autophagosomes. Immunity. (2007) 26:79–92. doi: 10.1016/j.immuni.2006.10.018. PMID: 17182262 PMC1805710

[45] Everts B Amiel E Huang SC Smith AM Chang CH Lam WY . Tlr-driven early glycolytic reprogramming via the kinases tbk1-ikkε supports the anabolic demands of dendritic cell activation. Nat Immunol. (2014) 15:323–32. doi: 10.1038/ni.2833. PMID: 24562310 PMC4358322

[46] Ho NI Camps MGM Verdoes M Münz C Ossendorp F . Autophagy regulates long-term cross-presentation by murine dendritic cells. Eur J Immunol. (2021) 51:835–47. doi: 10.1002/eji.202048961. PMID: 33349928 PMC8248248

[47] Jantsch J Chakravortty D Turza N Prechtel AT Buchholz B Gerlach RG . Hypoxia and hypoxia-inducible factor-1 alpha modulate lipopolysaccharide-induced dendritic cell activation and function. J Immunol (Baltimore Md: 1950). (2008) 180:4697–705. doi: 10.4049/jimmunol.180.7.4697. PMID: 18354193

[48] Kim J Kundu M Viollet B Guan KL . Ampk and mtor regulate autophagy through direct phosphorylation of ulk1. Nat Cell Biol. (2011) 13:132–41. doi: 10.1038/ncb2152. PMID: 21258367 PMC3987946

[49] Li J Lin Q Shao X Li S Zhu X Wu J . Hif1α-bnip3-mediated mitophagy protects against renal fibrosis by decreasing ros and inhibiting activation of the nlrp3 inflammasome. Cell Death Dis. (2023) 14:200. doi: 10.1038/s41419-023-05587-5. PMID: 36928344 PMC10020151

[50] Lampropoulou V Sergushichev A Bambouskova M Nair S Vincent EE Loginicheva E . Itaconate links inhibition of succinate dehydrogenase with macrophage metabolic remodeling and regulation of inflammation. Cell Metab. (2016) 24:158–66. doi: 10.1016/j.cmet.2016.06.004. PMID: 27374498 PMC5108454

[51] Oberkampf M Guillerey C . Mitochondrial reactive oxygen species regulate the induction of Cd8(+) T cells by plasmacytoid dendritic cells. (2018) 9:2241. doi: 10.1038/s41467-018-04686-8, PMID: 29884826 PMC5993805

[52] Bosteels C Neyt K Vanheerswynghels M van Helden MJ Sichien D Debeuf N . Inflammatory type 2 Cdcs acquire features of Cdc1s and macrophages to orchestrate immunity to respiratory virus infection. Immunity. (2020) 52:1039–1056.e9. doi: 10.1016/j.immuni.2020.04.005. PMID: 32392463 PMC7207120

[53] Beuneu H Deguine J Bouvier I Di Santo JP Albert ML Bousso P . Cutting edge: A dual role for type I Ifns during polyinosinic-polycytidylic acid-induced Nk cell activation. J Immunol (Baltimore Md: 1950). (2011) 187:2084–8. doi: 10.4049/jimmunol.1004210. PMID: 21810605

[54] Hurley HJ Dewald H Rothkopf ZS Singh S Jenkins F Deb P . Frontline science: Ampk regulates metabolic reprogramming necessary for interferon production in human plasmacytoid dendritic cells. J Leukoc Biol. (2021) 109:299–308. doi: 10.1002/jlb.3hi0220-130. PMID: 32640499

[55] Gill MA Bajwa G George TA Dong CC Dougherty II Jiang N . Counterregulation between the Fcepsilonri pathway and antiviral responses in human plasmacytoid dendritic cells. J Immunol (Baltimore Md: 1950). (2010) 184:5999–6006. doi: 10.4049/jimmunol.0901194. PMID: 20410486 PMC4820019

[56] Cella M Jarrossay D Facchetti F Alebardi O Nakajima H Lanzavecchia A . Plasmacytoid monocytes migrate to inflamed lymph nodes and produce large amounts of type I interferon. Nat Med. (1999) 5:919–23. doi: 10.1038/11360. PMID: 10426316

[57] Barr DP Belz GT Reading PC Wojtasiak M Whitney PG Heath WR . A role for plasmacytoid dendritic cells in the rapid Il-18-dependent activation of Nk cells following Hsv-1 infection. Eur J Immunol. (2007) 37:1334–42. doi: 10.1002/eji.200636362. PMID: 17407097 PMC2699043

[58] McGill J Van Rooijen N Legge KL . Il-15 trans-presentation by pulmonary dendritic cells promotes effector Cd8 T cell survival during influenza virus infection. J Exp Med. (2010) 207:521–34. doi: 10.1084/jem.20091711. PMID: 20212069 PMC2839152

[59] Greene TT Jo Y Chiale C Macal M Fang Z Khatri FS . Metabolic deficiencies underlie reduced plasmacytoid dendritic cell Ifn-I production following viral infection. Nat Commun. (2025) 16:1460. doi: 10.1038/s41467-025-56603-5. PMID: 39920132 PMC11805920

[60] Coenen I Jones AC White AA Takashima M Lee WR Wong MD . Reduced type-I interferon by plasmacytoid dendritic cells and asthma in school-aged children. Allergy. (2026) 81:109–20. doi: 10.1111/all.70005. PMID: 40799176 PMC12773650

[61] Delgoffe GM Kole TP Zheng Y Zarek PE Matthews KL Xiao B . The Mtor kinase differentially regulates effector and regulatory T cell lineage commitment. Immunity. (2009) 30:832–44. doi: 10.1016/j.immuni.2009.04.014. PMID: 19538929 PMC2768135

[62] Keating SE Zaiatz-Bittencourt V Loftus RM Keane C Brennan K Finlay DK . Metabolic reprogramming supports Ifn-Γ production by Cd56bright Nk cells. J Immunol (Baltimore Md: 1950). (2016) 196:2552–60. doi: 10.4049/jimmunol.1501783. PMID: 26873994

[63] Donnelly RP Loftus RM Keating SE Liou KT Biron CA Gardiner CM . Mtorc1-dependent metabolic reprogramming is a prerequisite for Nk cell effector function. J Immunol (Baltimore Md: 1950). (2014) 193:4477–84. doi: 10.4049/jimmunol.1401558. PMID: 25261477 PMC4201970

[64] Velásquez SY Killian D Schulte J Sticht C Thiel M Lindner HA . Short term hypoxia synergizes with interleukin 15 priming in driving glycolytic gene transcription and supports human natural killer cell activities. J Biol Chem. (2016) 291:12960–77. doi: 10.1074/jbc.M116.721753. PMID: 27129235 PMC4933215

[65] Schafer JR Salzillo TC Chakravarti N Kararoudi MN Trikha P Foltz JA . Education-dependent activation of glycolysis promotes the cytolytic potency of licensed human natural killer cells. J Allergy Clin Immunol. (2019) 143:346–358.e6. doi: 10.1016/j.jaci.2018.06.047. PMID: 30096390

[66] Wang J Zhou Y Zhang H Hu L Liu J Wang L . Pathogenesis of allergic diseases and implications for therapeutic interventions. Signal Transduct Target Ther. (2023) 8:138. doi: 10.1038/s41392-023-01344-4. PMID: 36964157 PMC10039055

[67] Helou DG Shafiei-Jahani P Lo R Howard E Hurrell BP Galle-Treger L . Pd-1 pathway regulates Ilc2 metabolism and Pd-1 agonist treatment ameliorates airway hyperreactivity. Nat Commun. (2020) 11:3998. doi: 10.1038/s41467-020-17813-1. PMID: 32778730 PMC7417739

[68] Facciabene A Peng X Hagemann IS Balint K Barchetti A Wang LP . Tumour hypoxia promotes tolerance and angiogenesis via Ccl28 and T(Reg) cells. Nature. (2011) 475:226–30. doi: 10.1038/nature10169. PMID: 21753853

[69] Li S Zhang Y Tong H Sun H Liao H Li Q . Metabolic regulation of immunity in the tumor microenvironment. Cell Rep. (2025) 44:116463. doi: 10.1016/j.celrep.2025.116463. PMID: 41129316

[70] Jameson G Walsh A Woods R Batten I Murphy DM Connolly SA . Human tissue-resident Nk cells in the lung have a higher glycolytic capacity than non-tissue-resident Nk cells in the lung and blood. PNAS. (2024) 121:e2412489121. doi: 10.1073/pnas.2412489121. PMID: 39378091 PMC11494342

[71] Yoshida K Morishima Y Ishii Y Mastuzaka T Shimano H Hizawa N . Abnormal saturated fatty acids and sphingolipids metabolism in asthma. Respir Invest. (2024) 62:526–30. doi: 10.1016/j.resinv.2024.04.006. PMID: 38640569

[72] Barnes PJ Baker J Donnelly LE . Autophagy in asthma and chronic obstructive pulmonary disease. Clin Sci (London England: 1979). (2022) 136:733–46. doi: 10.1042/cs20210900. PMID: 35608088 PMC9131388

[73] Guo F Hao Y . Asthma susceptibility gene Ormdl3 promotes autophagy in human bronchial epithelium. (2022) 66:661–70. doi: 10.1165/rcmb.2021-0305OC, PMID: 35353673 PMC9163638

[74] Mintern JD Macri C Chin WJ Panozza SE Segura E Patterson NL . Differential use of autophagy by primary dendritic cells specialized in cross-presentation. Autophagy. (2015) 11:906–17. doi: 10.1080/15548627.2015.1045178. PMID: 25950899 PMC4502655

[75] Zhang H Bosch-Marce M Shimoda LA Tan YS Baek JH Wesley JB . Mitochondrial autophagy is an Hif-1-dependent adaptive metabolic response to hypoxia. J Biol Chem. (2008) 283:10892–903. doi: 10.1074/jbc.M800102200. PMID: 18281291 PMC2447655

[76] Moffatt MF Kabesch M Liang L Dixon AL Strachan D Heath S . Genetic variants regulating Ormdl3 expression contribute to the risk of childhood asthma. Nature. (2007) 448:470–3. doi: 10.1038/nature06014. PMID: 17611496

[77] Siow D Sunkara M Dunn TM Morris AJ Wattenberg B . Ormdl/serine palmitoyltransferase stoichiometry determines effects of Ormdl3 expression on sphingolipid biosynthesis. J Lipid Res. (2015) 56:898–908. doi: 10.1194/jlr.M057539. PMID: 25691431 PMC4373746

[78] Xie T Liu P Wu X Dong F Zhang Z Yue J . Ceramide sensing by human Spt-Ormdl complex for establishing sphingolipid homeostasis. Nat Commun. (2023) 14:3475. doi: 10.1038/s41467-023-39274-y. PMID: 37308477 PMC10261145

[79] Oyeniran C Sturgill JL Hait NC Huang WC Avni D Maceyka M . Aberrant Orm (yeast)-like protein isoform 3 (Ormdl3) expression dysregulates ceramide homeostasis in cells and ceramide exacerbates allergic asthma in mice. J Allergy Clin Immunol. (2015) 136:1035–1046.e6. doi: 10.1016/j.jaci.2015.02.031. PMID: 25842287 PMC4591101

[80] Mushaben EM Kramer EL Brandt EB Khurana Hershey GK Le Cras TD . Rapamycin attenuates airway hyperreactivity, goblet cells, and Ige in experimental allergic asthma. J Immunol (Baltimore Md: 1950). (2011) 187:5756–63. doi: 10.4049/jimmunol.1102133. PMID: 22021618 PMC3221931

[81] Turnquist HR Raimondi G Zahorchak AF Fischer RT Wang Z Thomson AW . Rapamycin-conditioned dendritic cells are poor stimulators of allogeneic Cd4+ T cells, but enrich for antigen-specific Foxp3+ T regulatory cells and promote organ transplant tolerance. J Immunol (Baltimore Md: 1950). (2007) 178:7018–31. doi: 10.4049/jimmunol.178.11.7018. PMID: 17513751

[82] Lawless SJ Kedia-Mehta N Walls JF McGarrigle R Convery O Sinclair LV . Glucose represses dendritic cell-induced T cell responses. Nat Commun. (2017) 8:15620. doi: 10.1038/ncomms15620. PMID: 28555668 PMC5459989

[83] Thwe PM Pelgrom LR Cooper R Beauchamp S Reisz JA D'Alessandro A . Cell-intrinsic glycogen metabolism supports early glycolytic reprogramming required for dendritic cell immune responses. Cell Metab. (2017) 26:558–567.e5. doi: 10.1016/j.cmet.2017.08.012. PMID: 28877459 PMC5657596

[84] Xu YD Cheng M Mao JX Zhang X Shang PP Long J . Clara cell 10 (Cc10) protein attenuates allergic airway inflammation by modulating lung dendritic cell functions. (2024) 81:321. doi: 10.1007/s00018-024-05368-z, PMID: 39078462 PMC11335244

[85] Stonier SW Schluns KS . Trans-presentation: A novel mechanism regulating Il-15 delivery and responses. Immunol Lett. (2010) 127:85–92. doi: 10.1016/j.imlet.2009.09.009. PMID: 19818367 PMC2808451

[86] Zhu L Xie X Zhang L Wang H Jie Z Zhou X . Tbk-binding protein 1 regulates Il-15-induced autophagy and Nkt cell survival. Nat Commun. (2018) 9:2812. doi: 10.1038/s41467-018-05097-5. PMID: 30022064 PMC6052109

[87] Okamura H Tsutsi H Komatsu T Yutsudo M Hakura A Tanimoto T . Cloning of a new cytokine that induces Ifn-gamma production by T cells. Nature. (1995) 378:88–91. doi: 10.1038/378088a0. PMID: 7477296

[88] Chaix J Tessmer MS Hoebe K Fuséri N Ryffel B Dalod M . Cutting edge: Priming of Nk cells by Il-18. J Immunol (Baltimore Md: 1950). (2008) 181:1627–31. doi: 10.4049/jimmunol.181.3.1627. PMID: 18641298 PMC5154249

[89] Hasnat MA Cheang I Dankers W Lee JP Truong LM Pervin M . Investigating immunoregulatory effects of myeloid cell autophagy in acute and chronic inflammation. Immunol Cell Biol. (2022) 100:605–23. doi: 10.1111/imcb.12562. PMID: 35652357 PMC9542007

[90] Murai H Okazaki S Hayashi H Kawakita A Hosoki K Yasutomi M . Alternaria extract activates autophagy that induces Il-18 release from airway epithelial cells. Biochem Biophys Res Commun. (2015) 464:969–74. doi: 10.1016/j.bbrc.2015.05.076. PMID: 26032499 PMC4915742

[91] Reiners KS Dassler J Coch C Pogge von Strandmann E . Role of exosomes released by dendritic cells and/or by tumor targets: Regulation of Nk cell plasticity. Front Immunol. (2014) 5:91. doi: 10.3389/fimmu.2014.00091. PMID: 24639679 PMC3945280

[92] Viaud S Terme M Flament C Taieb J André F Novault S . Dendritic cell-derived exosomes promote natural killer cell activation and proliferation: A role for Nkg2d ligands and Il-15ralpha. PloS One. (2009) 4:e4942. doi: 10.1371/journal.pone.0004942. PMID: 19319200 PMC2657211

[93] Liu H Pan M Liu M Zeng L Li Y Huang Z . Lactate: A rising star in tumors and inflammation. Front Immunol. (2024) 15:1496390. doi: 10.3389/fimmu.2024.1496390. PMID: 39660139 PMC11628389

[94] ElKashef S Ahmad SE Soliman YMA Mostafa MS . Role of Microrna-21 and Microrna-155 as biomarkers for bronchial asthma. Innate Immun. (2021) 27:61–9. doi: 10.1177/1753425920901563. PMID: 31986951 PMC7780351

[95] Dosil SG Lopez-Cobo S Rodriguez-Galan A Fernandez-Delgado I Ramirez-Huesca M Milan-Rois P . Natural killer (Nk) cell-derived extracellular-vesicle shuttled Micrornas control T cell responses. eLife. (2022) 11:e76319. doi: 10.7554/eLife.76319. PMID: 35904241 PMC9366747

[96] Krawczyk CM Holowka T Sun J Blagih J Amiel E DeBerardinis RJ . Toll-like receptor-induced changes in glycolytic metabolism regulate dendritic cell activation. Blood. (2010) 115:4742–9. doi: 10.1182/blood-2009-10-249540. PMID: 20351312 PMC2890190

[97] Cramer T Yamanishi Y Clausen BE Förster I Pawlinski R Mackman N . Hif-1alpha is essential for myeloid cell-mediated inflammation. Cell. (2003) 112:645–57. doi: 10.1016/s0092-8674(03)00154-5. PMID: 12628185 PMC4480774

[98] Jedlička M Feglarová T Janstová L Hortová-Kohoutková M Frič J . Lactate from the tumor microenvironment - a key obstacle in Nk cell-based immunotherapies. Front Immunol. (2022) 13:932055. doi: 10.3389/fimmu.2022.932055. PMID: 36330529 PMC9623302

[99] Torres-Rosas R Yehia G Peña G Mishra P del Rocio Thompson-Bonilla M Moreno-Eutimio MA . Dopamine mediates vagal modulation of the immune system by electroacupuncture. Nat Med. (2014) 20:291–5. doi: 10.1038/nm.3479. PMID: 24562381 PMC3949155

[100] Cheng M Shang PP Wei DD Long J Zhang X Wu QL . Modulation of lung Cd11b(+) dendritic cells by acupuncture alleviates Th2 airway inflammation in allergic asthma. (2025) 20:67. doi: 10.1186/s13020-025-01119-9, PMID: 40405264 PMC12100888

[101] Nurwati I Muthmainah M Huda KN . Acupuncture for asthma: its potential significance in clinical practice. Med Acupunct. (2020) 32:272–9. doi: 10.1089/acu.2020.1443. PMID: 33101571 PMC7583338

[102] Owen MR Doran E Halestrap AP . Evidence that metformin exerts its anti-diabetic effects through inhibition of complex 1 of the mitochondrial respiratory chain. Biochem J. (2000) 348:607–14. doi: 10.1042/bj3480607. PMID: 10839993 PMC1221104

[103] Bharath LP Agrawal M McCambridge G Nicholas DA Hasturk H Liu J . Metformin enhances autophagy and normalizes mitochondrial function to alleviate aging-associated inflammation. Cell Metab. (2020) 32:44–55.e6. doi: 10.1016/j.cmet.2020.04.015. PMID: 32402267 PMC7217133

[104] Wu TD Fawzy A Akenroye A Keet C Hansel NN McCormack MC . Metformin use and risk of asthma exacerbation among asthma patients with glycemic dysfunction. J Allergy Clin Immunol In Pract. (2021) 9:4014–4020.e4. doi: 10.1016/j.jaip.2021.07.007. PMID: 34293503 PMC8675235

[105] Yang CC Tseng KL Lin YJ Weng SF Ho CH . Metformin use despite contraindications in metformin-associated lactic acidosis. Medicine. (2025) 104:e45197. doi: 10.1097/md.0000000000045197. PMID: 41189139 PMC12537172

[106] Trompette A Gollwitzer ES Yadava K Sichelstiel AK Sprenger N Ngom-Bru C . Gut microbiota metabolism of dietary fiber influences allergic airway disease and hematopoiesis. (2014) 20:159–66. doi: 10.1038/nm.3444, PMID: 24390308

[107] Rezk N-E Alshamy A Shehta M Morsy NE Alnahas M . Changing the airway pH: is it helping asthma control? (2021) 70:48–53. doi: 10.4103/ejcdt.ejcdt_98_20

[108] Teach SJ Gill MA Togias A Sorkness CA Arbes SJ Calatroni A . Preseasonal treatment with either omalizumab or an inhaled corticosteroid boost to prevent fall asthma exacerbations. J Allergy Clin Immunol. (2015) 136:1476–85. doi: 10.1016/j.jaci.2015.09.008. PMID: 26518090 PMC4679705

[109] Corren J Katelaris CH Castro M Maspero JF Ford LB Halpin DMG . Effect of exacerbation history on clinical response to dupilumab in moderate-to-severe uncontrolled asthma. Eur Respir J. (2021) 58:2004498. doi: 10.1183/13993003.04498-2020. PMID: 34266940 PMC8551561

[110] Contoli M Papi A . Effects of anti-IL-5 on virus-induced exacerbation in asthma. Light and shadow. Am J Respir Crit Care Med. (2019) 199:410–1. doi: 10.1164/rccm.201809-1684ED. PMID: 30265568

[111] Sabogal Piñeros YS Bal SM van de Pol MA Dierdorp BS Dekker T Dijkhuis A . Anti-IL-5 in mild asthma alters rhinovirus-induced macrophage, B-cell, and neutrophil responses (Material). A placebo-controlled, double-blind study. Am J Respir Crit Care Med. (2019) 199:508–17. doi: 10.1164/rccm.201803-0461OC. PMID: 30192638

[112] Fernandez MI Heuzé ML Martinez-Cingolani C Volpe E Donnadieu MH Piel M . The human cytokine TSLP triggers a cell-autonomous dendritic cell migration in confined environments. Blood. (2011) 118:3862–9. doi: 10.1182/blood-2010-12-323089. PMID: 21772055

[113] Hartung T . Look back in anger - what clinical studies tell us about preclinical work. Altex. (2013) 30:275–91. doi: 10.14573/altex.2013.3.275. PMID: 23861075 PMC3790571

[114] Møller SH Wang L Ho PC . Metabolic programming in dendritic cells tailors immune responses and homeostasis. (2022) 19:370–83. doi: 10.1038/s41423-021-00753-1, PMID: 34413487 PMC8891341

[115] Weichhart T Hengstschläger M Linke M . Regulation of innate immune cell function by mTOR. Nat Rev Immunol. (2015) 15:599–614. doi: 10.1038/nri3901. PMID: 26403194 PMC6095456

[116] Zeki AA Yeganeh B Kenyon NJ Post M Ghavami S . Autophagy in airway diseases: a new frontier in human asthma? Allergy. (2016) 71:5–14. doi: 10.1111/all.12761. PMID: 26335713 PMC4715640

[117] Karp T Faiz A van Nijnatten J Kerstjens HAM Boudewijn I Kraft M . Nasal epithelial gene expression identifies relevant asthma endotypes in the ATLANTIS study. Thorax. (2024) 79:905–14. doi: 10.1136/thorax-2023-221230. PMID: 39009441

[118] Weathington N O'Brien ME Radder J Whisenant TC Bleecker ER Busse WW . BAL cell gene expression in severe asthma reveals mechanisms of severe disease and influences of medications. Am J Respir Crit Care Med. (2019) 200:837–56. doi: 10.1164/rccm.201811-2221OC. PMID: 31161938 PMC6812436

[119] Vieira Braga FA Kar G Berg M Carpaij OA Polanski K Simon LM . A cellular census of human lungs identifies novel cell states in health and in asthma. Nat Med. (2019) 25:1153–63. doi: 10.1038/s41591-019-0468-5. PMID: 31209336

[120] Travaglini KJ Nabhan AN Penland L Sinha R Gillich A Sit RV . A molecular cell atlas of the human lung from single-cell RNA sequencing. Nature. (2020) 587:619–25. doi: 10.1038/s41586-020-2922-4. PMID: 33208946 PMC7704697

